# Topologically selective islet vulnerability and self-sustained downregulation of markers for β-cell maturity in streptozotocin-induced diabetes

**DOI:** 10.1038/s42003-020-01243-2

**Published:** 2020-09-30

**Authors:** Max Hahn, Pim P. van Krieken, Christoffer Nord, Tomas Alanentalo, Federico Morini, Yan Xiong, Maria Eriksson, Jürgen Mayer, Elena Kostromina, Jorge L. Ruas, James Sharpe, Teresa Pereira, Per-Olof Berggren, Erwin Ilegems, Ulf Ahlgren

**Affiliations:** 1 Umeå Centre for Molecular Medicine, Umeå University, Umeå, Sweden; 2 The Rolf Luft Research Center for Diabetes and Endocrinology, Karolinska Institutet, Stockholm, Sweden; 3 Centre for Genomic Regulation (CRG), The Barcelona Institute of Science and Technology, Barcelona, Spain; 4 Department of Physiology and Pharmacology, Biomedicum, Karolinska Institutet, Stockholm, Sweden; 5 European Molecular Biology Laboratory, Barcelona, Spain

**Keywords:** Experimental models of disease, 3-D reconstruction, Optical imaging, Diabetes, Mechanisms of disease

## Abstract

Mouse models of Streptozotocin (STZ) induced diabetes represent the most widely used preclinical diabetes research systems. We applied state of the art optical imaging schemes, spanning from single islet resolution to the whole organ, providing a first longitudinal, 3D-spatial and quantitative account of β-cell mass (BCM) dynamics and islet longevity in STZ-treated mice. We demonstrate that STZ-induced β-cell destruction predominantly affects large islets in the pancreatic core. Further, we show that hyperglycemic STZ-treated mice still harbor a large pool of remaining β-cells but display pancreas-wide downregulation of glucose transporter type 2 (GLUT2). Islet gene expression studies confirmed this downregulation and revealed impaired β-cell maturity. Reversing hyperglycemia by islet transplantation partially restored the expression of markers for islet function, but not BCM. Jointly our results indicate that STZ-induced hyperglycemia results from β-cell dysfunction rather than β-cell ablation and that hyperglycemia in itself sustains a negative feedback loop restraining islet function recovery.

## Abstract (web summary)

Hahn, van Krieken et al. provide a quantitative account of β-cell mass dynamics and islet longevity in mice treated with Streptozotocin (STZ). They find that STZ-induced hyperglycemia primarily results from β-cell dysfunction rather than its ablation. This study provides insights into how the most widely used preclinical diabetes model works.

## Introduction

The pancreas controls multiple homeostatic functions through the activities of its constituent exocrine acinar and endocrine islet cells. The endocrine component of the gland is organized into the islets of Langerhans (constituting ≈1–2% of the total pancreatic mass), which are scattered in great numbers throughout the exocrine parenchyma. Amongst the different endocrine cells of the islets, the insulin producing β-cells are the most prominent type both in humans and mice. Given its role in glucose metabolism, the pancreas is a key organ in the etiology of diabetes mellitus, a worldwide epidemic disease characterized by hyperglycemia. Although new ways of classifying diabetes were recently presented^1^, the disease is usually categorized in two major forms. In type 1 diabetes the islet β-cells are destroyed by an autoimmune attack causing absolute insulin insufficiency. The other major form, type 2 diabetes, is characterized by insulin resistance and insulin secretion deficiency, in many cases leading to an eventual reduction in β-cell mass (BCM) defined as a relative decrease in the total number of β-cells within the pancreas.

The study of pathophysiology, underlying mechanisms, and the treatment of diabetes requires well-characterized animal models that reflect main aspects of the human disease. Streptozotocin (STZ) is a hydrophilic glucose analogue synthesized by *Streptomyces achromogenes* that is particularly toxic to pancreatic β-cells^2,3^. STZ can pass the β-cell membrane through the low affinity GLUT2 transporter, which in rodents is the major mediator of glucose into β-cells and as such a vital part of the β-cell glucose sensing mechanism. STZ may contribute to the induction of diabetes by various mechanisms (recently reviewed by Radenkovic et al.^4^), including by alkylation-induced DNA damage and subsequent necrosis of β-cells^2^. Depending on the mode of administration, STZ may mimic different features of diabetes disease progression. Administered as a single high dose (SHD) it generates a simple model of severe β-cell destruction and hyperglycemia, while a regimen of multiple low doses (MLD) is suggested to generate a model of induced insulitis^5,6^. As such, STZ treatment may mimic primary pathophysiological features of human diabetes including pancreatic β-cell destruction, decreased insulin production, hyperglycemia, body weight loss, and other diabetes-induced complications^7^. Due to its simplicity, high incidence of diabetes and predictable time course of disease progression, STZ treatment of rodents^8^ has become the perhaps most extensively used animal model for diabetes research (≈60,000 articles in relation to “Streptozotocin diabetes” listed in PubMed as of June 2020). Models of STZ-induced diabetes have been used in a variety of research undertakings including e.g., studies of β-cell preservation, endocrine cell transdifferentiation, development of noninvasive β-cell imaging techniques, and immune cell infiltration^9–12^. Although the frequent use of STZ-treated mice as models of diabetes has led to numerous stereological 2D sampling assessments of BCM, studies describing (i) the effect of STZ treatment over time on individual islets in vivo or (ii) the BCM distribution in the entire pancreas subject to STZ treatment are essentially lacking. In this report, we use a range of state-of-the-art optical imaging techniques to refine our understanding of the BCM dynamics and its topological distribution in the murine pancreas subject to SHD and MLD of STZ administration. We additionally detail the pancreas-wide expression level of GLUT2 in relation to STZ-induced hyperglycemia at single islet resolution and quantitatively address the effect of STZ treatment on markers for β-cell function and maturity. Jointly, the presented data is likely to affect how STZ models are used and interpreted with regard to the relationship between (functional) BCM and the development of hyperglycemia.

## Results

### β-cell destruction by STZ primarily affects large islets

**Fig. 1:**
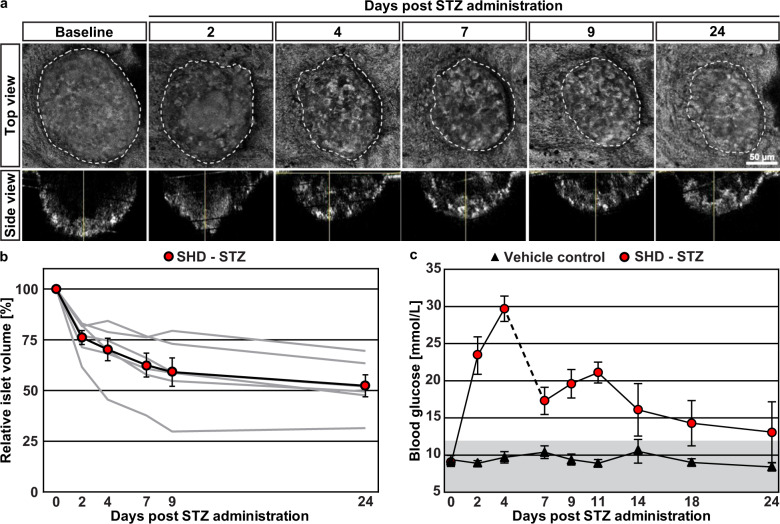
Streptozotocin-induced β-cell destruction kinetics revealed by longitudinal in vivo imaging of islet grafts. **a** Individual islets transplanted into the ACE were imaged by in vivo confocal microscopy at several time points. Backscatter imaging allowed to capture volumetric data of islet grafts^16^. Following a baseline assessment 1 month after transplantation, host mice received a single high dose of Streptozotocin (SHD-STZ, 200 mg/kg) to induce β-cell ablation. Displayed are maximum projection images of confocal stacks from the same islet graft over time. **b** Quantification of islet graft volumes for each time point reveals a gradual decrease following β-cell destruction. Data are displayed per mouse (gray lines) and as the average (black line, *n* = 6 mice). **c** Blood glucose values of STZ and sham treated mice over time. To prevent excessive weight loss as a consequence of hyperglycemia, the SHD-STZ group received ~100 additional islets in the non-transplanted eye at day 6. Gray shading in (**c**) represents normoglycemic levels, as defined by blood glucose concentration ≤12 mmol/L. Error bars represent SEM.

Although STZ has been demonstrated to cause β-cell death at sufficiently high dosages, the precise kinetics of β-cell destruction in relation to the emergence of hyperglycemia has not been studied in detail. Using the cornea as a natural body window and the anterior chamber of the eye (ACE) as a transplantation site, various aspects of β-cell biology may be studied longitudinally by in vivo confocal microscopy^13–17^. Using this platform, we previously determined that interventions affecting pancreatic islet mass are mirrored in intraocular islet grafts while sham treatments do not alter graft size^14,17^. While the complete range of islet sizes existing in situ in the pancreas cannot be represented by islets engrafted in the ACE (Supplementary Fig. 1), due to intrinsic limitations of collagenase-based digestion protocols for islet isolation, morphological changes can be determined with high temporal resolution. To be able to study β-cell destruction over time in relation to hyperglycemia, we monitored islet morphology in the ACE of syngeneically transplanted C57BL/6 mice by confocal microscopy and measured glycaemia before and after STZ treatment. As illustrated in Fig. 1a, intraperitoneal administration of a SHD of STZ led to a progressive reduction in islet graft volume. Quantitative analysis at each time point indicated that STZ administration reduced engrafted islet sizes to approximately half of their initial volume and that the destruction of β-cells chiefly occurred during the first week post-treatment (Fig. 1b). As expected, while mice administered vehicle remained normoglycemic, STZ administration rapidly resulted in a hyperglycemic phenotype (Fig. 1c). Already at day 2 post-STZ administration, mice were unable to maintain normoglycemia in spite of the substantial islet volume remaining at this time point.

**Fig. 2:**
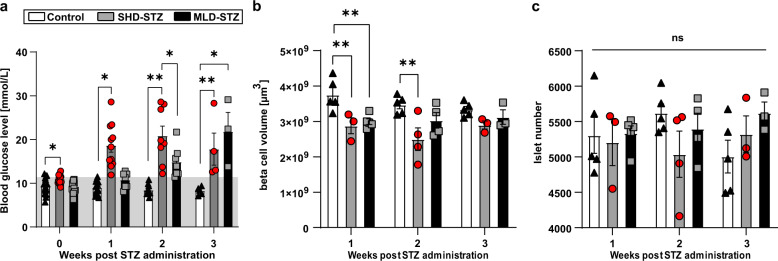
Mice with STZ-induced hyperglycemia display a modest reduction in BCM and unchanged islet numbers. **a** Blood glucose levels following SHD-STZ and MLD-STZ administration. *n* = 15 (week 0), *n* = 15 (week 1), *n* = 10 (week 2), and *n* = 5 (week 3). **b** Average BCM (defined here as β-cell volume, see text) in entire pancreata from hyperglycemic STZ-treated mice compared to untreated controls. Given the tomographic method of detection, BCM here corresponds to the volume of islet β-cells. **c** Average islet number in entire pancreata from hyperglycemic STZ-treated mice compared to untreated controls. In (**b**, **c**) *n* = 5 in controls at 1–3 weeks, *n* = 3 at 1 and 3 weeks and *n* = 4 at 2 weeks in SHD-STZ, *n* = 5 at 1 week, *n* = 4 at 2 weeks and *n* = 3 at 3 weeks in MLD-STZ. Gray shade in (**a**) represent normoglycemic levels, as defined by blood glucose concentration ≤12 mmol/L. Error bars represent SEM. * represents *P* ≤ 0.05, ** represents *P* ≤ 0.01.

To corroborate the results obtained from our ACE-platform and to assess how STZ induces β-cell destruction in the pancreas on the whole organ level (i.e., including the complete range of islets with maintained topological information), we next turned to optical projection tomography (OPT) imaging^18^, enabling assessments of individual islet β-cell volumes, and their spatial 3D coordinates throughout the volume of intact pancreata^19–22^. We performed two modes of STZ administration, SHD-STZ and MLD-STZ, to additionally investigate possible differences in the outcome of these procedures on the extent and/or kinetics of β-cell destruction. To this end, pancreata were collected before, 1, 2, and 3 weeks after administration of a SHD (150 mg/kg) or MLD (50 mg/kg once per day for 5 consecutive days) of STZ. Untreated mice were used as control group for OPT assessments, as no significant differences in BCM (*p* = 0.5472), islet number (*p* = 0.2513), or blood glucose levels (*p* = 0.06326 and *p* = 0.3045 at 3 weeks, SHD vehicle and MLD vehicle control respectively) compared to mice receiving an intraperitoneal injection of vehicle were detected (see Supplementary Fig. 2 and Methods section). As depicted in Fig. 2a, both SHD and MLD administration resulted in hyperglycemia (defined as blood glucose concentration > 12 mmol/L). However, in contrast to mice in the SHD group, no MLD-STZ-treated animals displayed elevated glucose levels a week after the first injection (blood glucose levels for all individual animals incorporated in the study are displayed in Supplementary Fig. 3). OPT-based overall BCM measurements from entire pancreata of overt diabetic mice demonstrated a significant, although relatively modest, BCM reduction in SHD-STZ diabetic mice in the first 2 weeks following STZ administration (Fig. 2b, *p* = 0.008 and *p* = 0.0014 at 1 and 2 weeks respectively), paralleled with the emergence of STZ-induced cellular apoptosis within islets (Supplementary Fig. 4). In contrast, a mild and significant reduction in BCM was only observed 1 week after STZ administration in MLD-STZ mice (Fig. 2b, *p* = 0.0078,). The total islet count did not show any significant differences between pancreata from untreated control mice and from hyperglycemic mice of either STZ administration procedure (Fig. 2c, SHD-STZ: *p* = 0.9545, *p* = 0.1616 and *p* = 0.6193 at 1, 2, and 3 weeks respectively, MLD-STZ: *p* = 0.9938, *p* = 0.7537 and 0.1773 at 1, 2, and 3 weeks respectively). Quantitative analysis of OPT data from the splenic, duodenal and gastric lobes of SHD-STZ and MLD-STZ showed that a substantial portion of the islet β-cells remained following STZ administration, regardless of their anatomical location (Supplementary Fig. 5).

**Fig. 3:**
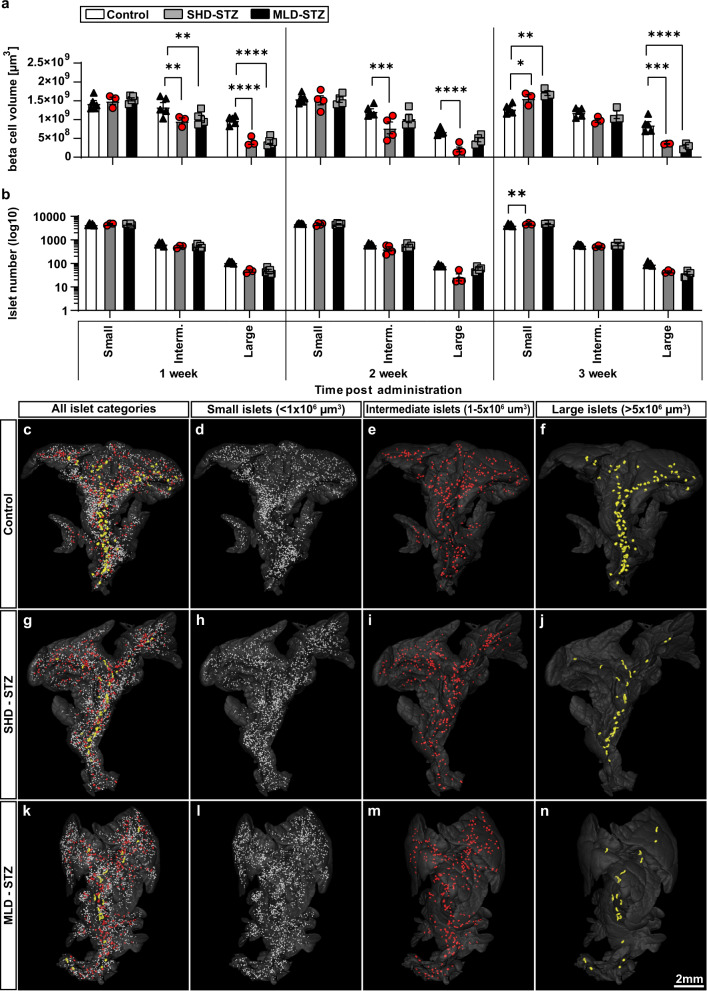
STZ-induced β-cell mass reduction is primarily attributed to reduction of large islets. **a**, **b** Graphs displaying the average volume (**a**) and number (**b**) of islets falling within arbitrarily selected size categories of small (<1 × 10^6^ µm^3^), medium (1–5 × 10^6^ µm^3^) and large (>5 × 10^6^ µm^3^) islets of Langerhans of hyperglycemic mice at 1, 2, and 3 weeks post-administration of SHD-STZ (Gray) MLD-STZ (Black) administration compared to untreated control (White) at 1, 2, and 3 weeks post-STZ administration, respectively. **c**–**n** Representative iso-surface rendered OPT images (splenic lobe only) of control pancreata (**c**–**f**) and 3 weeks post SHD-STZ (**g**–**j**) and MLD-STZ (**k**–**n**) administration. Islet β-cell volumes have been pseudo colored to delineate small (<1 × 10^6^ µm^3^) (**d**, **h**, **l**, white), medium (**e**, **i**, **m**, red, 1–5 × 10^6^ µm^3^), and large (**f**, **j**, **n**, yellow, >5 × 10^6^ µm^3^) islets. In **a**, **b** *n* = 5 in controls at 1–3 weeks, *n* = 3 at 1 and 3 weeks and *n* = 4 at 2 weeks in SHD-STZ, *n* = 5 at 1 week, *n* = 4 at 2 weeks and *n* = 3 at 3 weeks in MLD-STZ. Error bars represent SEM. *, **, ***, and **** represent *P* ≤ 0.05, ≤0.01, ≤0.001, and ≤0.0001 respectively.

To further refine the above picture, we divided the individual islet β-cell volumes obtained from SHD-STZ and MLD-STZ hyperglycemic mice into three arbitrarily chosen size categories: small (<1 × 10^6^ µm^3^), intermediate (1–5 × 10^6^ µm^3^) and large (>5 × 10^6^ µm^3^) (Fig. 3). OPT-based quantification of insulin labeled β-cell volumes shows that the observed BCM reduction (in Fig. 2) could be attributed primarily to islets belonging to the large size category at all time points analyzed (1, 2, and 3 weeks) post SHD-STZ administration (Fig. 3a). A similar pattern was observed for MLD-STZ-treated mice both 2 and 3 weeks after the first STZ administration (no animal had developed hyperglycemia after 1 week, see Fig. 2a). When assessing the number of islets belonging to each size category, the most prominent reduction was similarly observed in the large size category (Fig. 3b). In C57BL/6 mice, the largest islets are predominantly located in the center of the primary (splenic, duodenal and gastric) lobes of the pancreas whereas the periphery is dominated by smaller islets^23,24^. The above picture was confirmed when we pseudo colored the islets based on their size categories (Fig. 3c–n, Supplementary Movies 1–3). A similar pattern was observed in all lobes when analyzed individually. Noteworthy, the BCM constituted by the small size category displayed a significant increase at 3 weeks post-STZ administration in both cohorts (*p* = 0.044 and *p* = 0.0013 for SHD-STZ and MLD-STZ respectively, Fig. 3a), possibly due to that islets belonging to the larger categories become included in the small category following their reduction in size. Jointly these results suggest that the BCM reduction in both SHD-STZ and MLD-STZ-induced hyperglycemia is characterized primarily by a reduction in the number and size of large islets.

### STZ administration results in disturbed islet appearance

**Fig. 4:**
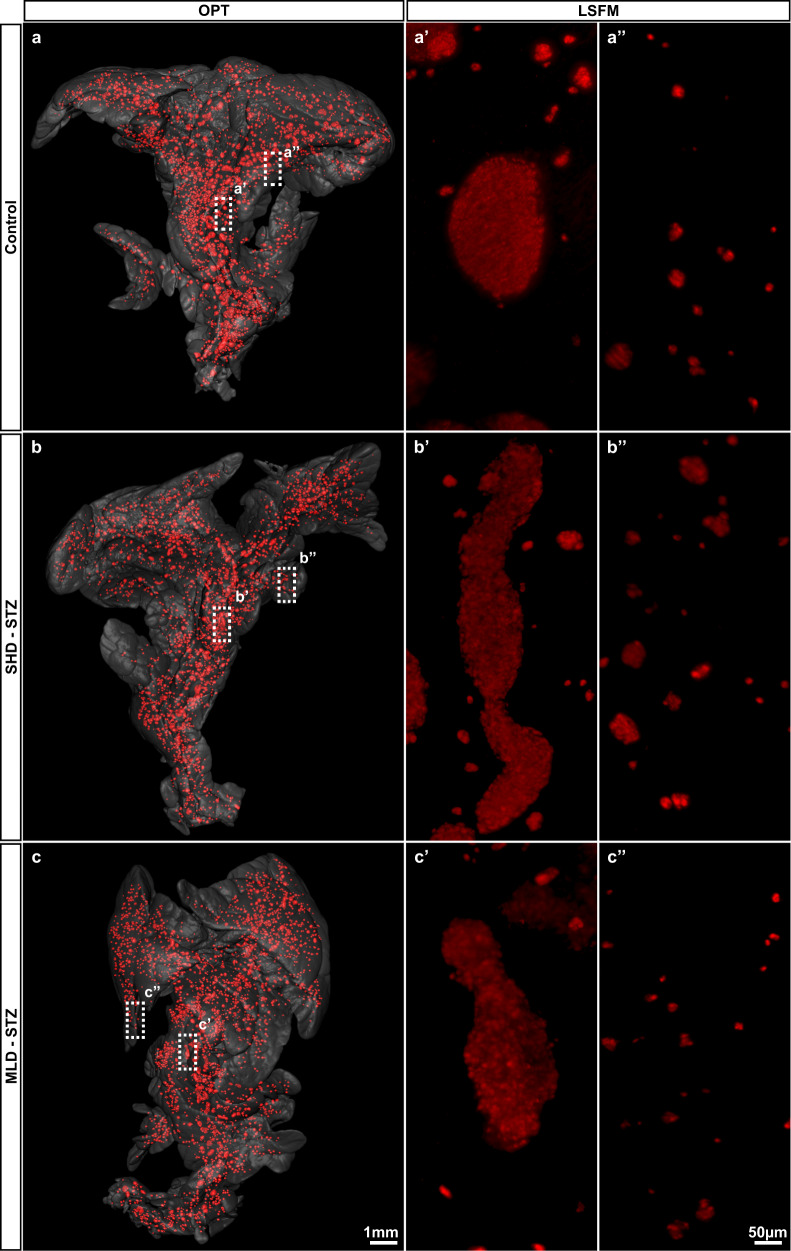
Large islets of STZ-treated mice display a disturbed and elongated morphology. **a**–**c** OPT images of splenic lobes of control (**a**), SHD-STZ (**b**) and MLD-STZ (**c**) pancreata 3 weeks post-STZ administration. Dashed boxes in (**a**–**c**) indicate the locations imaged by light sheet fluorescence microscopy (LSFM), displaying representative centrally-located large (a′–c′) and peripherally located small islets (a″–c″). LSFM images are shown as maximum intensity projections, from representative OPT samples (*n* = 3 pancreata per group). Note, samples were LSFM-scanned at different exposure times and are not intended for signal intensity comparisons.

Whereas the utility of OPT enables assessments of islet distributions throughout the volume of the murine pancreas, this whole organ imaging approach comes at the cost of limitations in resolution that could potentially influence the BCM quantified using this technique. In the current study, iso-surfaced volumes of 10 voxels or less were excluded from the data sets in order to avoid inclusion of artifacts from general noise (see Methods section). Although this translates to only about 7–8 β-cells (assuming these are spherical objects with a diameter of 10 µm) this could potentially result in a slight underestimation of the measured BCM. On the other hand, if STZ-mediated β-cell destruction would describe a scattered pattern within the islets, i.e., if the normal, essentially continuous, body of centrally-located β cells in the mouse islet would be “broken up”, non-insulin expressing regions could potentially be incorporated into the calculated islet β-cell volume and therefore lead to an overestimation of the measured BCM. With the aim of complementing the assessments performed by OPT with higher resolution data sets, we performed light sheet fluorescence microscopy (LSFM) on central regions (harboring predominantly large islets) and peripheral regions (harboring predominantly small islets) of the very same samples that were analyzed by OPT. These investigations showed that islets both in SHD-STZ and MLD-STZ hyperglycemic mice presented a seemingly continuous body of β cells 3 weeks post-STZ administration (Fig. 4). Interestingly, islets belonging to the large category, in addition to being reduced in numbers, frequently displayed an elongated morphology in both SHD-STZ and MLD-STZ hyperglycemic mice (Fig. 4b′, c′, Supplementary Movies 4–6, see also Fig. 3j, n, Supplementary Movies 1–3, and sphericity analysis in Supplementary Fig. 6). With the exception of MLD-STZ at 1 week post-administration, similar changes in islet morphology were observed for hyperglycemic mice at all time points analyzed. By further examination of islet morphology on tissue sections labeled for insulin and glucagon, our data shows that islets of hyperglycemic mice (both SHD-STZ and MLD-STZ) displayed a core consisting chiefly of β-cells (Supplementary Fig. 7). However, in contrast to control mice, α-cells were intermingled with β-cells as opposed to being predominantly located to the periphery of the islets, in agreement with previous reports^25,26^. Interestingly, a substantial portion of the β-cells in SHD-STZ and MLD-STZ pancreata exhibited markedly increased insulin staining intensities as compared to β-cells of control mice (Supplementary Fig. 8), while α-cells did not display any apparent differences in signal intensities between the groups. With the exception of MLD-STZ at 1 week post-administration (for which no animals developed hyperglycemia), a similar increase in insulin staining intensities was observed for hyperglycemic mice at all time points analyzed. Notwithstanding the possibility that BCM in STZ-treated mice incorporated in the study may be slightly overestimated in the OPT assays, these results suggest that a substantial amount of β-cells remained in hyperglycemic mice. Further they provide evidence that STZ treatment may induce prominent changes in islet morphology and insulin staining intensities.

### STZ induces reduced GLUT2 expression and β-cell maturity

**Fig. 5:**
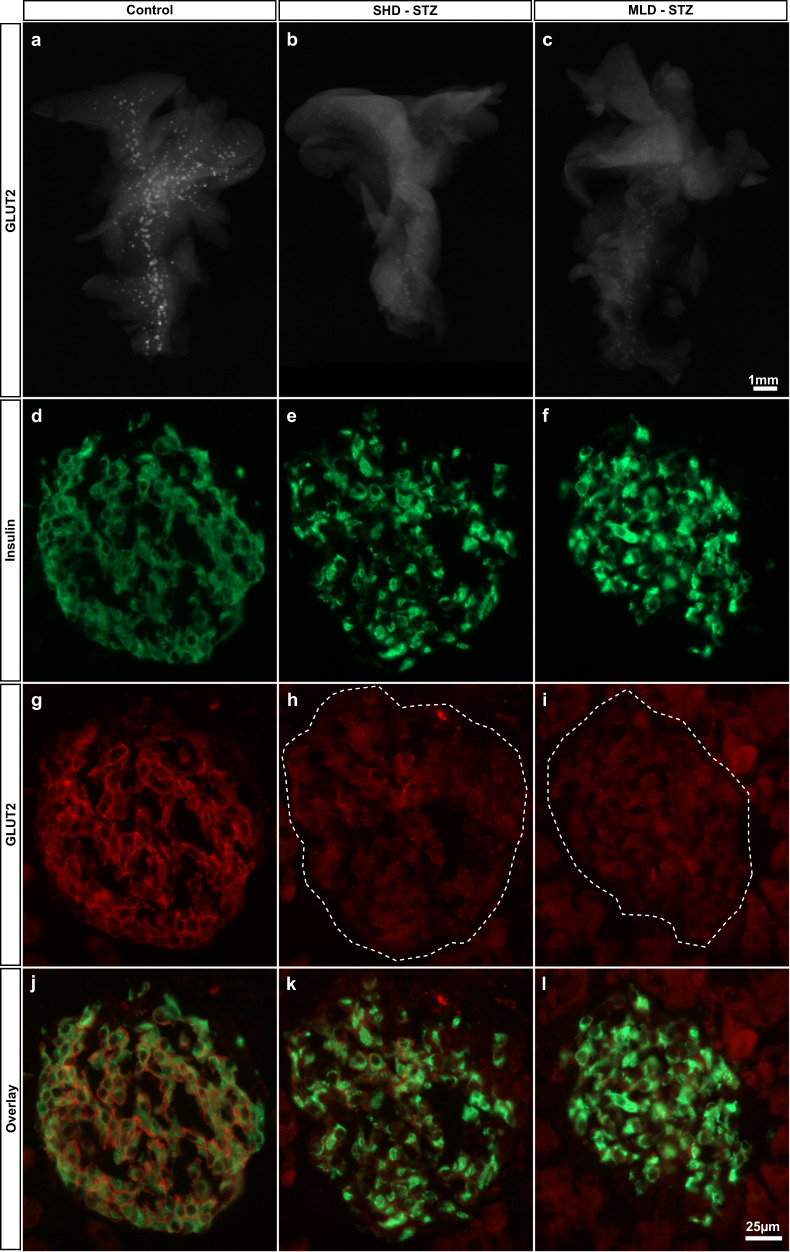
STZ administration results in a pancreas-wide downregulation of GLUT2 expression. **a**–**c** Representative OPT projection images showing GLUT2 expression in the intact splenic lobe of the pancreas of control (**a**), SHD-STZ (**b**) and MLD-STZ (**c**) hyperglycemic animals 3 weeks post-STZ administration. OPT images are representative of *n* = 5, 4, and 3 pancreata for control, hyperglycemic SHD-STZ, and hyperglycemic MLD-STZ conditions, respectively. **d**–**l** Immunohistochemical staining for insulin (**d**–**f**, green), GLUT2 (**g**–**i**, red), and insulin+GLUT2 (**j**–**l**) of pancreata from control (**d**, **g**, **j**), hyperglycemic SHD-STZ (**e**, **h**, **k**) and MLD-STZ (**f**, **i**, **l**) animals 3 weeks post-administration of STZ confirms the downregulation of GLUT2 in STZ-treated animals. In (**h**, **i**), the islet is indicated by a broken line.

Since impaired glucose stimulated insulin secretion has previously been associated with a markedly reduced expression of GLUT2 in diabetic rodents (see ref. ^27^ and references therein), and STZ administration has been shown to result in GLUT2 downregulation^28^, we next assessed the expression pattern of GLUT2 in the entire pancreas subject to SHD-STZ or MLD-STZ administration. As depicted in Fig. 5a–c, OPT projection data show an organ-wide downregulation of GLUT2 in both administration models. Immunohistochemical assessments on sections confirmed this picture and also pointed to an increased insulin staining intensity in STZ-treated mice (Fig. 5d–i). Similar differences in GLUT2 expression intensities between control and STZ-treated animals were observed for hyperglycemic mice at all time points analyzed (1–3 weeks post-STZ administration).

**Fig. 6:**
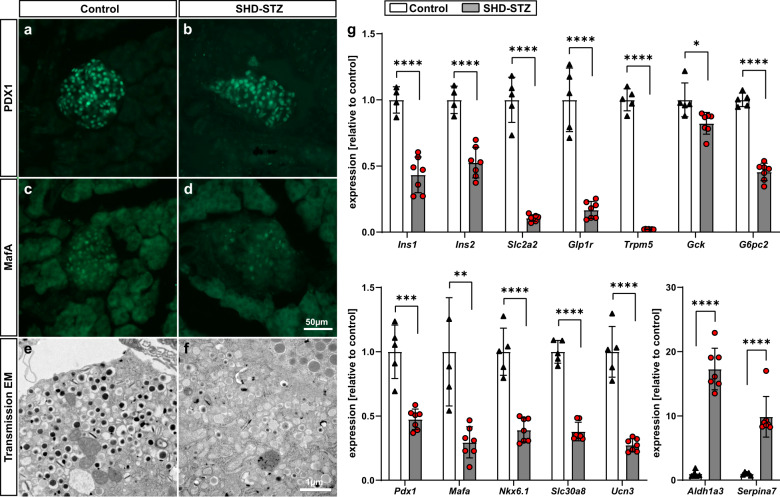
STZ administration leads to a decline in markers for β-cell function and maturity. **a**–**d** PDX1 (**a**, **b**) and MafA (**c**, **d**) immunohistochemistry on frozen pancreas sections showing islets from control (**a**, **c**) and from SHD-STZ (**b**, **d**) treated mice. Images are representative of three mice in each group. **e**, **f** Transmission electron microscopy images of islets isolated from control (**e**) and SHD-STZ mice (**f**), representative of islets from three mice per group. Note the apparent reduction of dense granules containing crystallized insulin in the islet from the SHD-STZ-treated animal. **g** Relative expression of genes associated to β-cell function, maturity and dedifferentiation determined by qRT-PCR of isolated islets from control and SHD-STZ mice (*n* = 5 and 7 mice for control and SHD-STZ, respectively). All samples were collected 3 weeks post-STZ administration. Error bars represent SD. *, **, ***, and **** represent *P* ≤ 0.05, ≤0.01, ≤0.001, and ≤0.0001 respectively.

To broaden our understanding of islet function after STZ treatment, we next investigated markers for β-cell function and identity 3 weeks after STZ administration. By qualitative immunohistochemistry we could detect the presence of markers for mature β-cells MafA and PDX1 both in pancreatic islets from control and SHD-STZ mice (Fig. 6a–d). However, and in agreement with previous studies^29,30^, ultrastructural assessment by transmission electron microscopy (TEM) on isolated islets indicated functional defects by the presence of immature insulin granules in place of typically dense granules containing crystallized insulin (Fig. 6e, f), which might account for the more transparent appearance of islets isolated from SHD-STZ mice. Gene expression analysis on freshly isolated islets displayed a reduction in markers for β-cell function and maturity (*Ins1*, *Ins2*, *Slc2a2*, *Glp1r*, *Trpm5*, *Gck*, *G6pc2*, *Pdx1*, *Mafa*, *Nkx6.1*, *Slc30a8*, and *Ucn3*) and an increase in the markers for β-cell dedifferentiation *Aldh1a3* and *Serpina7* in SHD-STZ as compared to control (Fig. 6g). The reduced expression of *Ins1* and *Ins2* corroborated a diminished islet insulin content in SHD-STZ as compared to control islets (Supplementary Fig. 9).

### Islet transplantation partly restores β-cell maturity

**Fig. 7:**
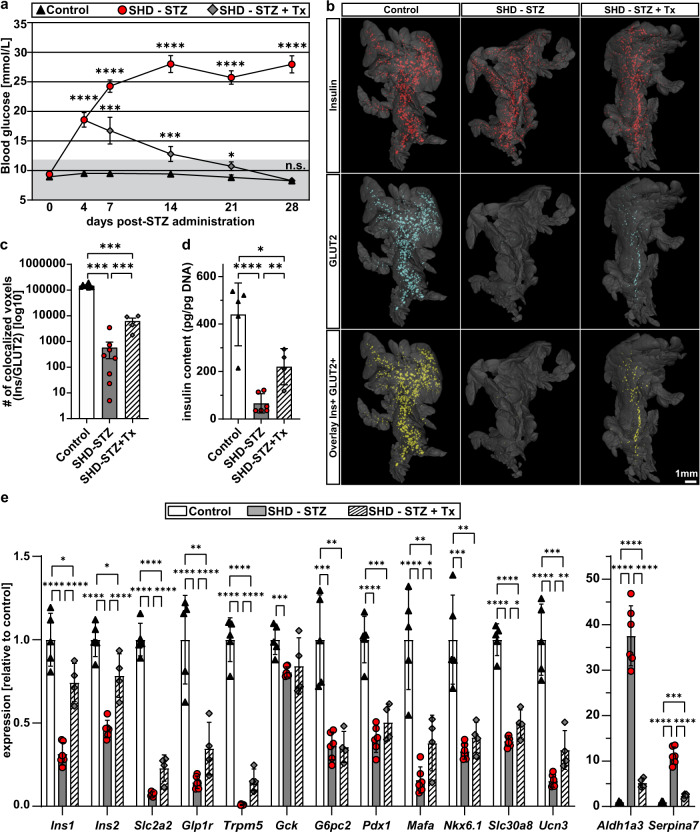
Pancreas-wide GLUT2 downregulation following STZ-induced hyperglycemia is partially recovered by islet transplantation into the ACE. **a** Blood glucose levels were measured during a period of 28 days after SHD-STZ (150 mg/kg) or vehicle injection. Four days after STZ administration, hyperglycemic mice were either syngeneically transplanted with ~100–150 islets into the ACE to restore normoglycemia (SHD-STZ + Tx), or left untransplanted (SHD-STZ). *n* (animals) = 13 for control, *n* = 15 for SHD-STZ, and *n* = 8 for SHD-STZ + Tx. Pancreata were harvested 28 days post-STZ administration for ex vivo OPT imaging or islet isolation. Gray shading represents normoglycemic levels, as defined by blood glucose concentration ≤ 12 mmol/L. **b** Analysis of GLUT2 expression in pancreatic islets reveals a partial recovery of GLUT2 expression in transplanted mice. Representative OPT renderings show pancreatic splenic lobes labeled for insulin (red), glucose transporter GLUT2 (blue), and overlay images (co-staining indicated by yellow color) in vehicle treated controls (*n* = 7), SHD-STZ (*n* = 8), and SHD-STZ + Tx (*n* = 4). **c** Quantitative assessment of the number of co-localizing voxels based on insulin and GLUT2 in control, SHD-STZ and SHD-STZ + Tx pancreata respectively. **d** Whole islet insulin content from isolated islets, normalized to DNA content. **e** Relative expression of genes associated to β-cell function, maturity and dedifferentiation determined by qRT-PCR on cDNA from isolated islets. **d**, **e** *n* = 5, 6, and 4 mice for vehicle control, SHD-STZ, SHD-STZ + Tx, respectively. Error bars represent SEM (**a**, **c**) or SD (**d**, **e**). *, **, ***, and ****represent *P* ≤ 0.05, ≤0.01, ≤0.001, and ≤0.0001 respectively.

In addition to enabling confocal microscopy based in vivo studies of islet biology, grafting of islets to the ACE provides an efficient means to decrease plasma glucose levels and to improve survival of STZ diabetic mice^31^. In order to delineate the effect of high glucose levels on pancreatic GLUT2 expression in STZ hyperglycemic mice, we transplanted ~100 islets into the ACE 4 days after administration of a single high dose of STZ (SHD-STZ + Tx). Pancreata were isolated and analyzed for BCM and GLUT2 expression 4 weeks post-administration, when islet transplantations had restored blood glucose to normal levels (Fig. 7a). As previously, we observed a reduction in BCM in the non-transplanted SHD-STZ-treated animals compared to control animals. Pancreata of SHD-STZ + Tx mice contained similar BCM compared to the non-transplanted group, indicating that the level of glycaemia had no effect on the pancreatic BCM, at least not within the timeframe of this experiment (Supplementary Fig. 10a). Compared to the dramatic downregulation of GLUT2 expression levels throughout the volume of the pancreas in the SHD-STZ animals, the intensity of GLUT2 staining was partly recovered in the transplanted mice (Fig. 7b, c, Supplementary Fig. 10e–g and Supplementary Movies 7–9). Indeed, co-localization analysis of voxels positive for GLUT2 and insulin indicated a more than tenfold increase in the pancreas of transplanted as compared to non-transplanted mice. Interestingly, while we also by OPT observed an increase in insulin staining intensities in pancreatic islets from the SHD-STZ as compared to control, this difference seemingly disappeared after transplantation (Supplementary Fig. 11). To investigate more in detail changes occurring to pancreatic islet cells after transplantation, we proceeded with islet isolations. Qualitatively, islets from mice administrated STZ had a more transparent appearance and their β-cells contained immature secretory granules, a property which partially reverted following transplantation (Supplementary Fig. 12). Interestingly, the STZ-induced decrease in gene expression of markers for β-cell function and maturity, as well as in islet insulin content, partially recovered in SHD-STZ + Tx pancreatic islets (Fig. 7d, e). The most striking change was however revealed by the drastic reduction in the expression of *Aldh1a3* and *Serpina7* in SHD-STZ + Tx islets, thereby evidencing the major involvement of hyperglycemia in β-cell dedifferentiation. Enquiring into β-cell proliferation, which has been previously observed to be of higher rate in islets from mice administrated STZ^32,33^, we could see by the expression levels of *Ccna2*, *Ccnb1*, *Ccnb2*, and *Ki67* that it has reversed in the SHD-STZ + Tx group to levels similar to control mice (Supplementary Fig. 13), demonstrating the essential implication of blood glucose levels on β-cell proliferation and on the regulation of BCM.

Jointly, in the timeframe of this study, these results demonstrate that restoring normoglycemia after STZ-induced hyperglycemia partly reverses the downregulation of GLUT2 in pancreatic islets and of markers for β-cell function and maturity without restoring BCM.

## Discussion

In this report, we provide detailed longitudinal and whole organ 3D data, in vivo and ex vivo, on the effect of STZ on islet longevity. Destruction of β-cells is often considered as the primary cause of hyperglycemia in models of STZ-induced diabetes, however our whole organ data suggests that the reduction of BCM could be surprisingly moderate regardless of the mode of STZ administration. Hence processes other than β-cell destruction may constitute key determinative factors for the development of hyperglycemia subject to STZ administration. Upon administration, the short-lived STZ molecules enter β-cells via the low affinity glucose transporter GLUT2^3,34,35^. Soon thereafter, β-cell destruction occurs as a result of oxidative stress and DNA alkylation^36,37^. Although a substantial portion of the β-cells survive the initial effects of STZ, the surviving pool to a great extent becomes dysfunctional, resulting in a rise in blood glucose levels and subsequent glucose toxicity. Jointly, these processes could contribute to a negative spiral of self-sustained β-cell impairment, including an organ-wide downregulation of GLUT2. Indeed, although not the only factor of importance for β-cell function, the impairment of GLUT2-mediated glucose detection in itself is a major factor in the development of glucose intolerance and an indication of impaired β-cell function^38^.

To delineate the long-lasting effects of acute STZ toxicity from the negative impact of hyperglycemia on GLUT2 expression level it is necessary to lower blood glucose levels. If normoglycemia is restored, as in our study by transplanting islets to the ACE, the negative feedback loop is broken; GLUT2 is upregulated, and islet function may be recovered in situ. While GLUT2 levels did not reach levels similar to that of control mice, at least not at the time point of our study, our results demonstrate that glycemia by itself plays an important role in the modulation of GLUT2 expression levels. In support of this notion it was previously found that *db/db* mouse islets, isolated from their diabetic environment and transplanted to the kidney capsule in a nondiabetic environment, restored their GLUT2 expression levels. Conversely, *db/+* islets from a nondiabetic environment lost their GLUT2 expression when transplanted to a diabetic environment^39^. As indicated by the slower rise in blood glucose levels and the less severe reduction in BCM in the MLD-STZ model compared to SHD-STZ-treated mice, it is plausible that the foremost difference between MLD and SHD administration lies in the weighting of the above-mentioned processes. Namely, although both models are influenced by the same parameters, hyperglycemia resulting from the SHD administration regimen may include a greater element of acute STZ toxicity whereas MLD administration may reflect a more gradual development of glucose toxicity.

Enquiring into the destruction pattern at the single islet level, we observed an heterogenous insulin staining after STZ treatment with an overall increase in insulin staining intensities. This is in striking contrast with the decreased expression of *Ins1* and *Ins2*, as well as with a corresponding decrease in islet insulin content. This apparent discrepancy could possibly be explained at the ultrastructural level by the lack of proper crystallization of the insulin granules: one could hypothesize that insulin antibodies have less access to their epitope when insulin molecules are densely packed as water-insoluble zinc-insulin hexamer crystals^40^, and/or that in properly crystallized granules the excitation and emission of fluorescently-labeled secondary antibodies are locally impaired, “masked” by the dark and relatively large secretory granules (200–400 nm in diameter^41,42^).

The reduced opacity of secretory granules observed in islets from STZ-treated mice corroborates the reduced expression of ZnT8 (encoded by *Slc30a8*), a transporter essential for proper crystallization of insulin within granules^43,44^. This is also reflected by the more transparent appearance of islets isolated from STZ-treated mice, similar to what has been reported for islets isolated from ZnT8-deficient mice^43^, as insulin granule crystals are of major importance for the optical detection of β-cells by side-scattering of light^16,45^.

**Fig. 8:**
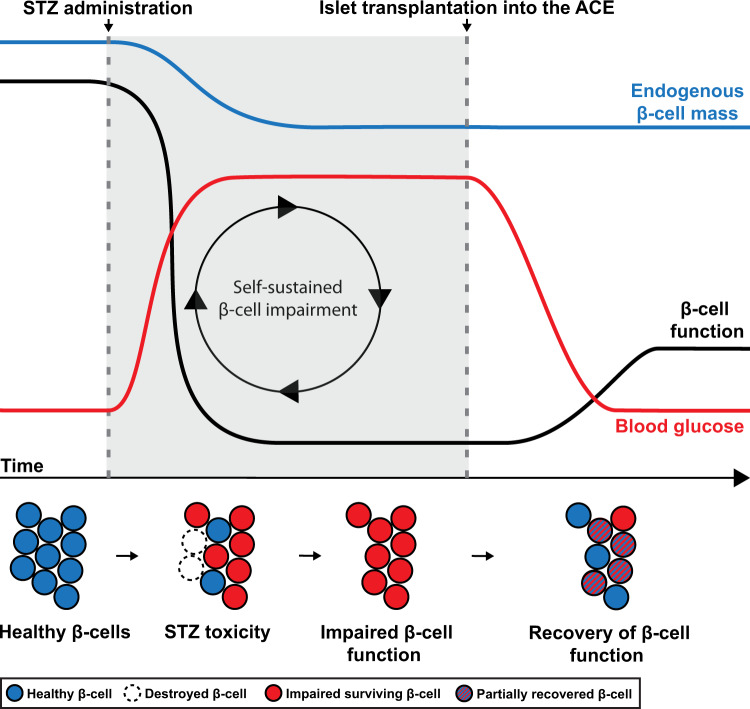
Schematic model outlining processes leading to development of STZ-induced diabetes in mice. According to our whole organ data, the reduction of BCM may be moderate in STZ diabetic mice subject to both SHD-STZ and MLD-STZ administration. Hence processes other than β-cell destruction are likely to constitute key determinative factors for the development of hyperglycemia, including a negative spiral of self-sustained β-cell impairment and a concomitant organ-wide downregulation of GLUT2 and other markers of β-cell function. By transplanting islets into the ACE, normoglycemia, and thereby endogenous β-cell functionality is partly restored (see “Discussion” for details).

As a reduction in GLUT2 levels alone is unlikely to cause drastic changes in islet function^46^, it is essential to assess the expression of additional genes that would be indicative of β-cell functional status. STZ administration has been demonstrated by gene array analyses to downregulate a wide range of genes vital for β-cell function 1 week after the start of MLD-STZ treatment, including the *Slc2a2* gene encoding GLUT2^26^. Our results indicate a similar downregulation of genes defining β-cell function and maturity both at 3 and at 4 weeks following SHD-STZ administration. In addition to the above-mentioned decrease in *Slc2a2*, *Ins1*, *Ins2*, and *Slc30a8*, we observed lower expression levels of *Glp1r*, *Trpm5*, *Gck*, *G6pc2*, *Ucn3*, and of the transcription factors *Pdx1*, *Mafa,* and *Nkx6.1*, essential markers of mature adult β-cells. The inferred reduced β-cell function was accompanied by the appearance of markers for β-cell dedifferentiation, *Aldh1a3*, and *Serpina7*^47^, a further indication of reduced islet function. Lowering the glycemic levels by transplanting islets into the ACE partially reversed the changes we observed in pancreatic islet gene expression. In particular, the markers for β-cell function *Ins1*, *Ins2*, *Slc2a2*, *Glp1r*, and *Trpm5* were clearly upregulated in islets from SHD-STZ + Tx as compared to non-transplanted SHD-STZ mice, alongside a major reduction in the expression of β-cell dedifferentiation markers. This suggests that hyperglycemia in itself sustains a dysfunctional endocrine pancreas function, and that pancreatic islets still have the capacity to reverse STZ-induced damage, at least to some extent (see Fig. 8). As our studies were not carried out at the single cell level but rather at individual islet level, we do not know whether (i) all cells within islets partially recover, or (ii) new mature β-cells are completing the overall function of individual islets without improving the surviving population of “damaged” β-cells within the islet. However, since the proliferation rate of islet cells in the recovered SHD-STZ + Tx mice was as low as in the control group, we can speculate that previously damaged cells have the ability to recover their function to a certain extent. Lineage tracing studies would be required to fully address this question at the single cell level.

At the entire pancreas level, we observed a relatively modest reduction in BCM regardless of the STZ administration model. The amount of β-cell destruction previously reported in the literature varies greatly, as it depends on a large number of factors. In addition to the assessment methodologies in use (which usually extrapolates values from a non-exhaustive number of sampled islets), the outcome of STZ administration is influenced by the dose and mouse strain^48^, the mouse age (due to age-related changes in β-cell regenerative capacity^49^) and the obtained glycemic level. Unexpectedly, and contrarily to what we previously described for the transgenic RIP-DTR diabetes mouse model^17^, the extent to which BCM was reduced was revealed in our current study to be strongly dependent on initial islet size. Indeed, large islets appeared more sensitive to STZ administration in terms of reduction of β-cell volume as compared to small ones. This finding is supported by a previous observation made in the context of a smaller scale study^50^ (based on ≈200 islets per pancreas as opposed to the current study in which all islets of each pancreas—around 5500—were analyzed, in total encompassing around 330,000 islets of all size categories). In addition, we observed that the remaining large islets frequently exhibited noticeable changes in islet morphology (Fig. 4, Supplementary Fig. 6 and Supplementary Movies 4–6), possibly reflecting a more intense destruction of β-cells in these islets, which could be followed by a rearrangement dictated by interorgan pressure and/or partial regeneration along the centrally-located pancreatic ducts. An increasing body of evidence suggests that the islets of Langerhans are functionally heterogeneous with regard to developmental origin, topology, size, and function^51–56^. It is therefore conceivable that the topological differences in BCM reduction upon STZ administration in the current study reflect such differences. Although the reason for the observed heterogeneity in BCM reduction remains unclear, it is possible that the larger islets, which are predominantly located centrally in the gland in close vicinity to the main vessels, are exposed to a higher local dose of the administered STZ. Another hypothesis could be that the larger islets display greater functionality in vivo, i.e., this subpopulation would take up more glucose per cell as compared to the other islets in their periphery and would therefore similarly take up a larger amount of administrated STZ, leading to an increased destruction. Their partial destruction would therefore also lead to an important decrease in the whole endocrine pancreas function. The preferential susceptibility of large islets subject to STZ administration is however in sharp contrast to the pattern of β-cell loss observed in the Non-Obese Diabetic (NOD) mouse, which spontaneously develops autoimmune insulitis-mediated diabetes. In the NOD model, the dynamics of BCM destruction appears to describe a seemingly opposite pattern in that the smallest islets are the first to be destroyed, whereas the largest islets even display a compensatory growth potential^19^, thus providing evidence for clearly different mechanisms affecting changes in BCM. Regardless of the mechanistic reason(s) behind these observations, our data may influence future (and previous) studies of STZ-diabetic animals in which the spatial dynamics of BCM is of importance for the evaluation of results. Importantly, as previously stated the largest islets of the mouse pancreas are located centrally in the gland^23,24^ (see also Fig. 3). Hence, the total loss of β-cell area may easily be overestimated if not all regions of the gland, including the peripheral regions, are represented proportionally, a task which is rarely performed (and difficult to execute), when conducting stereological assessments of BCM of the murine pancreas^23^. Our findings additionally suggest that models of STZ-induced diabetes are not optimal models of drastic β-cell loss for the evaluation of novel noninvasive imaging approaches.

To conclude, our data uncovers a size-dependent heterogenous destruction of murine islets following STZ administration, which might be reflective of a specific pool of pancreatic islets holding a predominant role in the overall endocrine pancreas function. Importantly, our data on the plasticity of GLUT2 expression level and on overall improvement in β-cell maturity following islet transplantation showcases the importance of glucose level normalization for a potential functional recovery of surviving β-cells in the framework of diabetes treatment.

## Methods

### Chemicals

All chemicals were from Sigma-Aldrich unless otherwise specified.

### Animals and STZ treatment

Male C57BL/6J mice were purchased from Charles River and housed at the Umeå Center for Comparative Biology and at the animal facilities at Karolinska Institutet, Stockholm. Animals had free access to water and, apart from 4–6 h before STZ treatment, standard rodent chow diet. STZ (Sigma) dissolved freshly in 0.1 M sodium citrate buffer (pH 4.5) was administered by intraperitoneal (i.p.) injection, either as a single high dose (SHD, 150–200 mg/kg) or as multiple low doses (MLD, 50 mg/kg once per day for 5 consecutive days). The selected STZ concentrations adhere to consensus in the literature in that SHD ranges from 100 to 200 mg/kg^57^ or follow the Jackson Laboratory MLD diabetes induction scheme for C57BL/6 mice. When comparing untreated control mice with mice receiving an intraperitoneal injection of vehicle (0.1 M sodium citrate buffer without STZ) no differences in BCM, islet number or blood glucose levels could be detected (Supplementary Fig. 2). For OPT assessments the control groups for STZ-treated mice were untreated, unless otherwise stated in the paper. Regular glucose measurements were performed from tail vein blood with OneTouch (LifeScan, USA, Figs. 2, S2, S3) or Accu-Chek (Roche, Figs. 1, 7) glucometers until the mice were euthanized for organ harvesting. All experiments were performed following the European Union guidelines for care and use of animals in research, and all procedures were approved by the Animal Review Board at the Court of Appeal of Northern Norrland and of Northern Stockholm.

### Islet isolation

Islets were isolated via ductal injection of 1 mg/ml collagenase P in Hanks’ Balanced Salt Solution (HBSS) buffered with HEPES (pH 7.4) and supplemented with 0.25% bovine serum albumin (BSA). Pancreata were thereafter digested in a 37 °C water bath for 22–24 min, islets were handpicked in ice-cold HBSS containing 0.5% BSA and either directly used (for islet gene expression, insulin content, and ultrastructural assessments), or cultured in RPMI-1640 medium supplemented with 10% fetal calf serum, L-glutamine (2 mM), penicillin (100 U/ml), and streptomycin (100 U/ml) (Thermo Fisher Scientific) for islet transplantations.

### Measurement of islet insulin content

Between 8 and 10 islets were lysed in M-PER protein extraction reagent (Thermo Fisher Scientific). Insulin content was assessed using AlphaLISA immunoassay (Perkin Elmer) and normalized to islet DNA content, measured using QuantIT Picogreen dsDNA kit (Thermo Fisher Scientific).

### Gene expression analysis

RNA from ~30–50 islets was prepared using RNeasy Micro Kit (QIAGEN) following the manufacturer’s instructions. Full-length cDNA was produced by reverse transcription using Superscript II (Thermo Fisher Scientific), amplified and purified as previously described^58^. Quantitative RT-PCR was performed using SYBR Green (Thermo Fisher Scientific) and a QuantStudio 5 system (Thermo Fisher Scientific). Concentrations of all samples were normalized prior to qRT-PCR experiments, all genes were normalized to TATA box binding protein (*Tbp*) and expressed relative to experimental controls. Primer sequences are listed in Supplementary Table 1.

### Islet transplantation and longitudinal confocal imaging

Islet transplantation and monitoring was performed as described^59^. In brief, islets were obtained from mice on a C57BL/6J background via collagenase digestion and handpicking. A small number (5–10) of islets were transplanted into the ACE of 8-week-old C57BL/6J mice under isoflurane anesthesia. One month after transplantation, confocal *z*-stacks of islet grafts (*n* ≥ 3 per mouse) were acquired by in vivo reflected light imaging^16^ using a Leica SP5 microscope system. To assess changes in islet size, graft-bearing mice underwent several imaging sessions at the indicated time points before and after STZ administration. Islet graft volumes were quantified using Fiji software as described^17^ and presented as the average per mouse (*n* = 3–12 grafts per eye). For the experiment shown in Fig. 1, SHD-STZ mice received an additional 75–100 islets in their non-transplanted eye 6 days post-treatment to prevent excessive weight loss as a consequence of hyperglycemia. In the experiments attempting to delineate the effect of hyperglycemia on GLUT2 expression and on β-cell function and maturity (Figs. 7, S10–S13), SHD-STZ animals with the highest blood glucose levels were syngeneically transplanted with 100–150 islets in the ACE 4 days post-STZ to revert hyperglycemia.

### Organ processing and optical projection tomography

Isolated pancreata (divided into the splenic, gastric, and duodenal lobular compartments^60^, see also Supplementary Fig. 5) were stained for insulin and processed for OPT imaging as previously described^23,61^. Briefly, the specimen were bleached to reduce autofluorescence, labeled with primary and secondary antibodies, mounted in a cylinder of low melting point agarose and made semi-transparent by clearing in a mixture of benzylalcohole:benzylebenzoate (BABB). All samples were blinded and randomized for OPT-processing after organ harvest. Antibodies used were guinea pig anti-insulin (DAKO A0564, dilution 1:500) and goat Alexa 594 anti-guinea pig (Molecular Probes, dilution 1:500). OPT scanning was performed as described using a Bioptonics 3001 OPT scanner (SkyScan, Belgium), applying a contrast limited adaptive histogram equalization (CLAHE) algorithm with a tile size 64 × 64 to the projection images^24,61^. For combined assessments of insulin and GLUT2 expression (Figs. 5, 7, S10), the pancreata were in addition to insulin labeled with rabbit anti-GLUT2 antibodies (Millipore, 07-1402-I, dilution 1:500), and secondary IRDye® 680RD goat antirabbit 680 (Licor, C80911-15, dilution 1:500). Scanning of these samples was performed in our in-house built Near Infrared-OPT setup^61^. For assessments of the effect of STZ on GLUT2 expression (intensity), all samples were imaged at the same exposure time and the CLAHE contrast normalization routine^24^ was not implemented. Note, for insulin and GLUT2 co-localization analysis, at the current resolution segmentation of labeled (co-expressing) cells/regions results in overlapping voxels despite insulin being expressed in the cytoplasm and GLUT2 in the cell membrane. For volumetric assessments of β-cell volumes, the CLAHE script was routinely implemented. Tomographic reconstruction was performed using the NRecon v1.6.9.18 software (Skyscan, Belgium). Insulin positive β-cell volumes were quantified by 3D iso-surfacing using the Imaris 9.3.1 software (Bitplane, UK). Islets were categorized into small (<1 × 10^6^ µm^3^), intermediate (1–5 × 10^6^ µm^3^), and large (>5 × 10^6^ µm^3^) as previous reported^19,21^. Iso-surfaced volumes of 10 voxels or less were excluded from the data sets obtained with the Bioptonics 3001 OPT scanner in order to avoid including artifacts from general noise or the occasional inclusion of artifacts (such as dust particles). The voxel filter was adapted accordingly for scans conducted in the Near Infrared-OPT instrument.

To compare sphericity of different islet sizes (Supplementary Fig. 6), the sphericity (1 being a perfect sphere) and volume of OPT-based iso-surfaced islets was measured by Imaris and extracted to generate ranges of islet volume categories. For easier understanding of islet sizes, approximal islet diameters were calculated from corresponding islet volumes with the assumption of a perfect sphere (*V* = 4πr^3^/3) and displayed as bin points.

For OPT quantitative intensity measurements (Supplementary Fig. 11), pancreatic splenic lobes of the control, SHD-STZ and SHD-STZ + Tx groups were processed and cleared the same way as for quantification of BCM. Subsequent Near Infrared-OPT images were generated using equal exposure times of the Insulin staining (filters used were Ex: HQ 565/30 nm, Em: HQ 620/60 nm, exposure time: 4000 ms) to compare insulin intensities between groups on the whole organ scale. A similar image processing pipeline to the insulin and GLUT2 co-localization assessment of identical processing parameters, not implementing the CLAHE contrast normalization technique was used to create tomographic sections to render insulin signal intensities as Maximum Intensity Projections in 3D in Imaris. By using identical parameters for the iso-surface thresholding algorithm in Imaris between groups, individual islets were segmented and the average insulin intensity within a closed surfaced islet was measured by the software. We then color coded the quantified and islet specific insulin intensities onto the surfaces to visualize organ-wide insulin intensities between groups. In addition, segmented and insulin intensity quantified islets were placed in islet volume categories to compare average insulin intensities between group with different islet sizes (Supplementary Fig. 11j).

### Light sheet fluorescence microscopy imaging

Representative pancreata that were OPT processed and imaged (see above), were reimaged by a LaVision biotech 2nd generation UltraMicroscope (LaVision BioTec GmbH, Germany) with ImSpectorPro Data Acquisition and Analysis Environment (version 5.0.164, LaVision BioTec GmbH, Germany). Characteristic islets with different sizes were targeted and located based on analyzed OPT data sets. The samples mounted in agarose were trimmed in BABB (for clearing method see refs. ^23,61^) to fit into the UltraMicroscope sample holder before image acquisition. The resultant image data were visualized and analyzed using Imaris 9.3.1 (Bitplane, UK).

### Immunohistochemistry

PFA-fixed pancreata were washed in PBS, dehydrated overnight in a 30% sucrose solution in deionised water, and frozen in Optimal Cutting Temperature (OCT, Tissue-Tek 4583). Cryo-sections with a thickness of 8 µm were immunolabelled according to standard protocol, images were taken with a Nikon Eclipse E800 microscope (Nikon) and analyzed using Imaris 9.3.1 (Bitplane, UK). Insulin staining intensity analysis was measured using Volocity (Perkin Elmer), data for each islet was presented as average signal intensity per pixel, corrected for background intensity. Primary antibodies were guinea pig anti-insulin (DAKO A0564, dilution 1:500), rabbit anti-GLUT2 (Millipore, 07-1402-I, dilution 1:500), rabbit anti-glucagon (ImmunoStar, 20076, dilution 1:500), rabbit anti Cleaved Caspase3 (Cell Signaling, 9661, dilution 1:500), rabbit anti MafA (Bethyl, A300-611A-3, dilution 1:250), and rabbit anti PDX-1 (Helena Edlund lab own stock, dilution 1:500). Secondary antibodies were goat Alexa Fluor® 594 anti-rabbit (Molecular Probes, A11012; dilution 1:500) and goat Alexa Fluor® 488 anti-guinea pig (Molecular Probes A11073, dilution 1:500).

### Transmission electron microscopy

Isolated islets were fixed in 2.5% glutaraldehyde +1% paraformaldehyde in 0.1 M phosphate buffer, pH 7.4. Specimens were rinsed in 0.1 M phosphate buffer, pH 7.4, and postfixed in 2% osmium tetroxide 0.1 M phosphate buffer, pH 7.4 at 4 °C for 2 h, stepwise dehydrated in ethanol followed by acetone and finally embedded in LX-112 (Ladd, Burlington, Vermont, USA). Ultrathin sections (~50–60 nm) were prepared using a Leica EM UC 7 ultramicrotome (Leica, Wien, Austria) and contrasted with uranyl acetate followed by lead citrate and examined in a Hitachi HT7700 transmission electron microscope (Hitachi Hightech, Japan) at 100 kV. Digital images were acquired using a Veleta camera (Olympus Soft Imaging Solutions, GmbH, Münster, Germany).

### Statistical analysis and reproducibility

Assumption of normality was tested within each group by normal distribution histogram. For all OPT-based assessments, i.e., beta cell volume, islet number, sphericity and OPT insulin intensities comparisons (Figs. 2, 3, S2, S5, S6, S10, S11) a two-way ANOVA with alpha = 0.05 and Tukey’s post hoc test of multiple comparisons was used. For comparing blood glucose levels, qRT-PCR expression levels, and Insulin content (Figs. 1, 6, 7, S9, S13) multiple *t* tests were applied. An ordinary one-way ANOVA was used to compare the number of colocalized voxels of insulin and GLUT2 (Fig. 7c), and for insulin staining intensities (Supplementary Fig. 8). Considered statistical significance was set to *p* < 0.05. Statistical analyses were carried out in Microsoft Excel 2016 or Excel 365 and GraphPad Prism v8.4.2.

Immunohistochemical experimental findings on sections were repeated at least twice with reproducible results. Glucose measurements were always performed in duplicate. The reproducibility of OPT-based assessments of BCM has previously been established^19,21,23,24^.

OPT sample size calculation was performed based on previous studies on diabetic mouse models^19,21,23^. Note, by nature of the tomographic technique used, the generated data sets are, at least in theory, “absolute”, i.e., all islets of each pancreas are included in the data sets (translating to about 5000 islets of Langerhans per control pancreas). In contrast, commonly utilized stereological sampling techniques estimate islets mass, number etc. based on a limited number of islets (normally only a few hundred). For transplantation experiments, sample size was determined based on previously performed experiments^17^.

All samples were randomized after organ harvest for OPT-processing, OPT-image acquisition and data analysis. For transplantation experiments, all mice were randomly allocated into their experimental group except for the mice receiving curative transplantation in the “recovery experiment” (SHD-STZ + Tx). These mice had the highest blood glucose 4 days after STZ treatment and would likely have suffered too much weight loss during the duration of the experiment if left untransplanted.

All samples were blinded to the investigator after organ harvest for OPT analyses. For longitudinal in vivo imaging experiments, blinding was not possible as blood glucose and islet volume measurements were unequivocally different between STZ-treated and control mice.

### Reporting summary

Further information on research design is available in the Nature Research Reporting Summary linked to this article.

## Supplementary information

Supplementary information

Description of Additional Supplementary Files

Supplementary Data 1

Supplementary Movie 1

Supplementary Movie 2

Supplementary Movie 3

Supplementary Movie 4

Supplementary Movie 5

Supplementary Movie 6

Supplementary Movie 7

Supplementary Movie 8

Supplementary Movie 9

Reporting Summary

## Data Availability

The data (including OPT projection views, tomographic image stacks, and Light sheet fluorescence data) that support the findings of this study constitute around 3 Terabytes. This data (or parts thereof) is available from the corresponding author upon reasonable request. Individual data points corresponding to Figs. 1b, c, 2a–c, 3a, b, 6g, 7a, c–e are shown in Supplementary Data 1.

## Code availability

For NIR-OPT^61^ in-house generated image processing software was used, including scripts for (i) COM-AR (i.e., semiautomatic positioning of the samples at the axis of rotation)^62^, (ii) determination of post-alignment values, and for (iii) ref. ^62^ and CLAHE^24^. These are available upon request to the authors subject to a material transfer agreement.
