# Supplementary material for: Topologically selective islet vulnerability and self-sustained downregulation of markers for β-cell maturity in streptozotocin-induced diabetes
Source: Commun Biol. 2020 Sep 30;3:541. doi: 10.1038/s42003-020-01243-2 (PMC7527346; doi:10.1038/s42003-020-01243-2)
Supplement: Supplementary file 1 — Supplementary information [file 42003_2020_1243_MOESM1_ESM.pdf]

SUPPLEMENTARY FIGURES

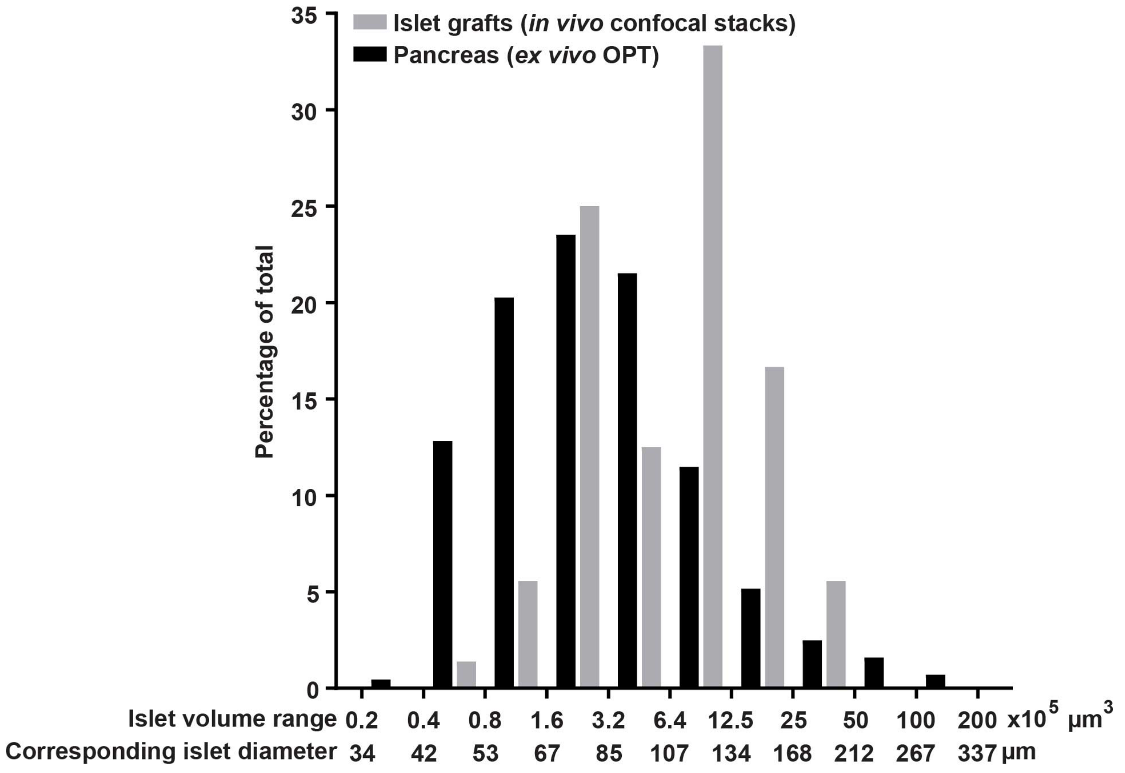

Supplementary Figure 1. Size distribution of islets in situ in the pancreas as compared to islets engrafted into the ACE. Histogram displaying the number of islets belonging to arbitrarily chosen size categories, shown as a percentage of the total number of islets. Black bars display OPT-based volumetric data from a representative mouse pancreas (n=5353 islets). Gray bars represent pooled data from the ACE imaged by confocal microscopy after intraocular transplantation (72 islets grafted and analyzed in 10 mice). For indicative purposes, corresponding islet diameters are shown below the x-axis, calculated assuming islets are spherical objects. Mice used for tissue collection and transplantation were 8 weeks of age and on a C57BL/6J genetic background. In vivo imaging of islets engrafted into the ACE was performed four weeks post-transplantation.

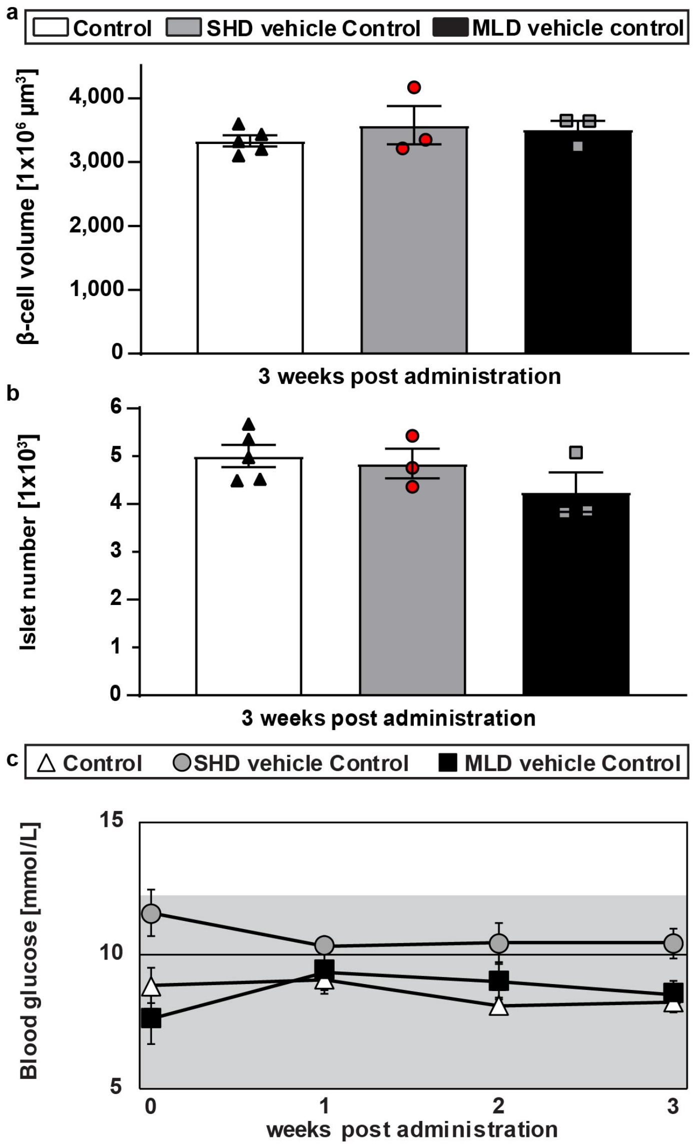

Supplementary Figure 2. BCM and islet number do not differ between vehicle controls and untreated controls. a, Average β-cell volumes determined by OPT ex vivo in untreated control, SHD vehicle control and MLD vehicle controls in C57BL/6 pancreata (n=3 for all groups). **b**, Average pancreatic islet numbers of the pancreata analyzed in (a). **c**, Blood glucose concentrations determined at indicated time points of the groups analyzed in (a, b). Gray shading represents normoglycemic levels, as defined by blood glucose concentration ≤ 12 mmol/L. Error bars represent SEM.

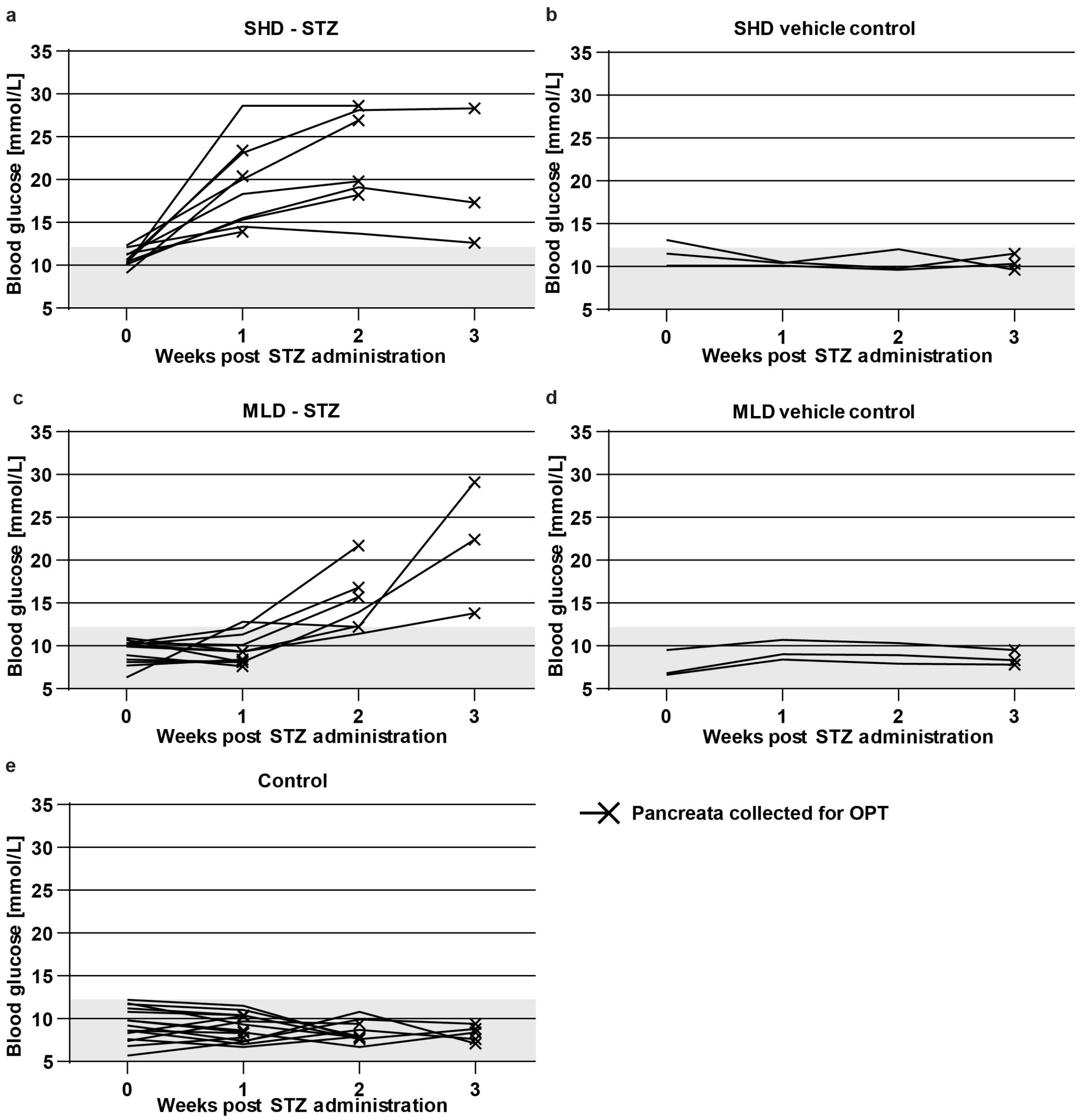

Supplementary Figure 3. Blood glucose levels for the individual mice included in the study (OPT assessments only). a-e, Individual animal blood glucose level at indicated timepoints for SHD-STZ (a), SHD vehicle control (b), MLD-STZ (c). MLD-vehicle control (d) and untreated control (e), respectively.

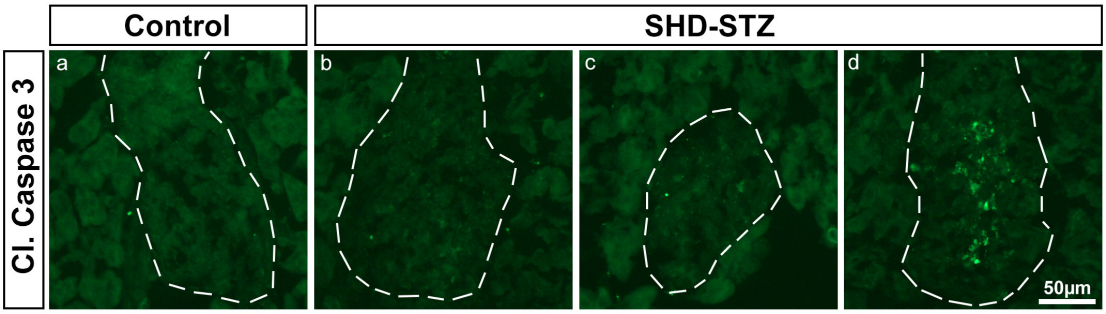

Supplementary Figure 4. Cellular apoptosis in islets of diabetic STZ-treated animals. a-d, Cleaved Caspase-3 stainings of islets from control (a) and SHD-STZ treated animals two weeks after STZ administration (b-d). Whereas control animals did not display cleaved Caspase-3 positive cells, islets of STZ treated animals displayed staining ranging from low to moderate. Stainings in (b-d) are from the same mouse and are representative of the heterogenous positive stainings observed for SHD-STZ diabetic mice between 1 and 3 weeks post STZ administration (n=6).

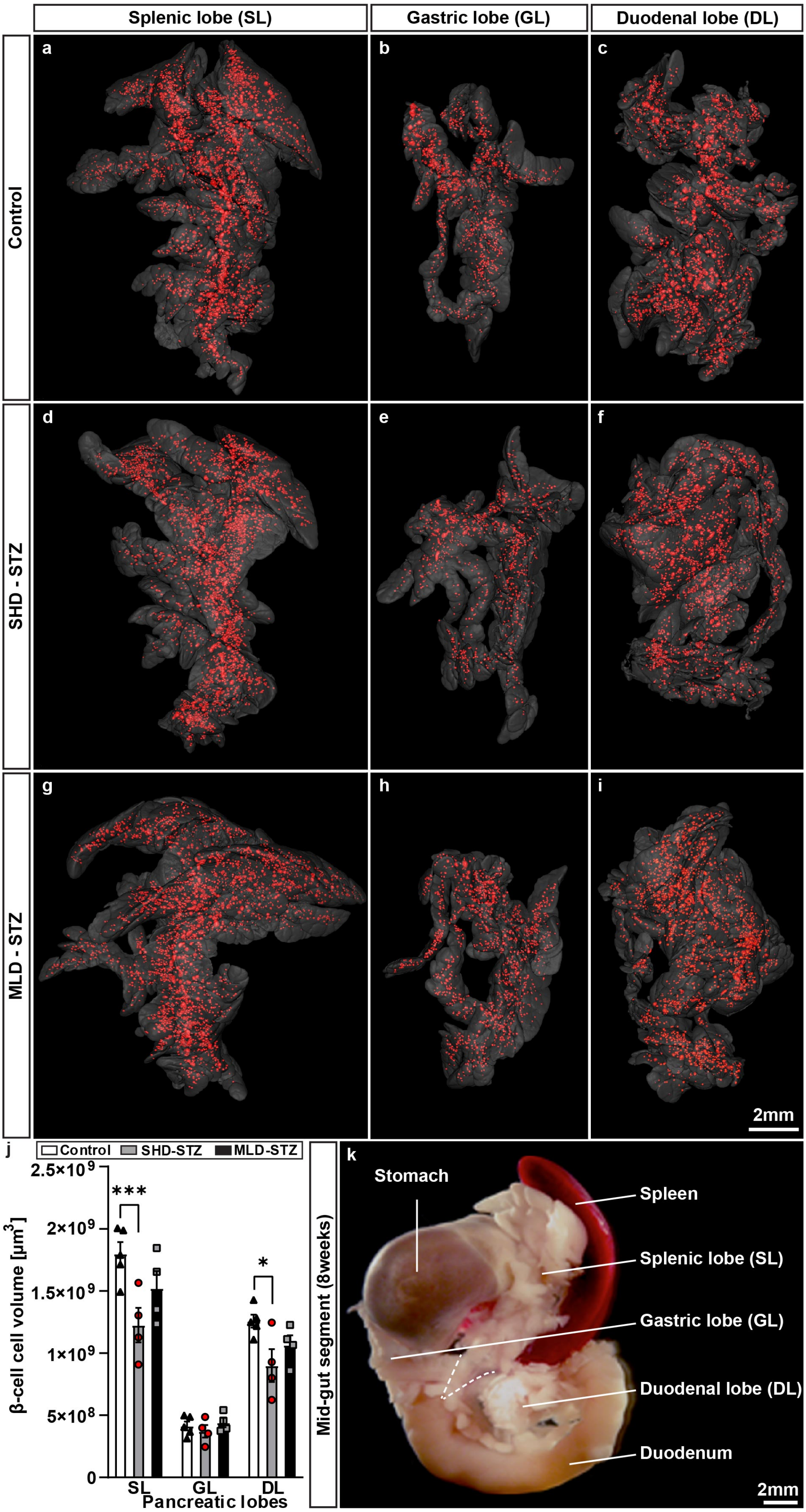

Splenic lobe (SL)
Gastric lobe (GL)
Duodenal lobe (DL)
Control
SHD - STZ
MLD - STZ
a
b
c
d
e
f
g
h
i
2mm
j
Control
SHD-STZ
MLD-STZ
2.5×10^9
2×10^9
1.5×10^9
1×10^9
5×10^8
0
β-cell cell volume [µm^3]
***
*
SL
GL
DL
Pancreatic lobes
k
Mid-gut segment (8weeks)
Stomach
Spleen
Splenic lobe (SL)
Gastric lobe (GL)
Duodenal lobe (DL)
Duodenum
2mm

Supplementary Figure 5. Remaining β-cell mass in the primary lobes of the pancreas subject to STZ treatment. a-i, Representative iso-surface rendered OPT images displaying the BCM distribution (red) in splenic (a, d, g), gastric (b, e, h) and duodenal lobes (c, f, i) of control (a-c), SHD-STZ (d-f) and MLD-STZ (g-i) animals at 2 weeks post-administration (stage at which the largest difference compared to control was observed, see Fig. 2). **j**, Average β-cell volumes in the respective primary lobes at the corresponding stage (n=5 in controls, n=4 in SHD-STZ and n=4 in MLD-STZ). **k**, Gut segment encompassing the pancreas, stomach, duodenum and spleen at 8 weeks of a C57BL/6 mouse illustrating the anatomical locations of the primary lobular compartments of the mouse pancreas (Hörnblad et al., PLoS One, 2013, separated by broken white line). Error bars represent SEM. * and ** represents P-values ≤ 0.05 and ≤ 0.01respectively.

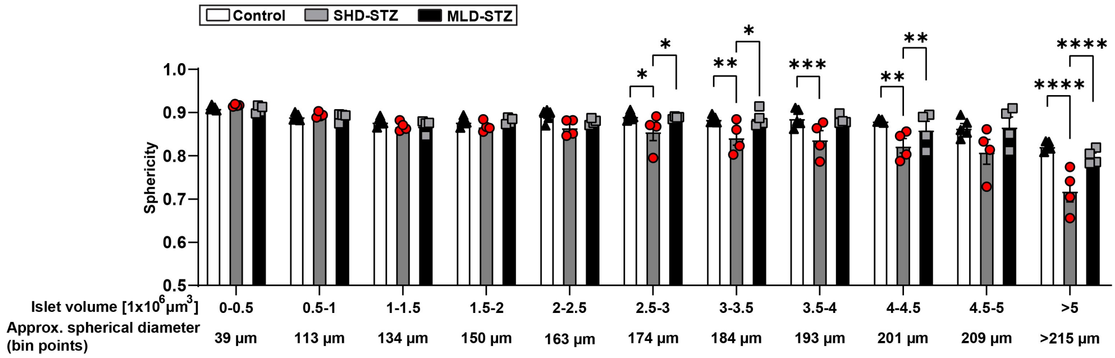

Supplementary Figure 6. Large islets display altered morphology following STZ administration. The graph displays OPT based sphericity assessments of iso-surfaced islets plotted against volume categories and their corresponding theoretical diameter (a perfect sphere corresponds to “1” on the y-axis). Islet sphericity is reduced in islets belonging to the larger size categories in islets of STZ treated animals. Measurements are performed 2-weeks post SHD-STZ and MLD-STZ treatment (similar results were obtained at 1- and 3-weeks post STZ treatment). Each symbol in the graph corresponds to pancreata from individual animals, controls (n=5, 28,108 islets), SHD-STZ (n=4, 20,154 islets) and MLD-STZ (n=4, 21,595 islets). Error bars represent SEM. *, **, *** and **** represent $P \leq 0.05$, ≤ 0.01, ≤ 0.001 and ≤ 0.0001 respectively.

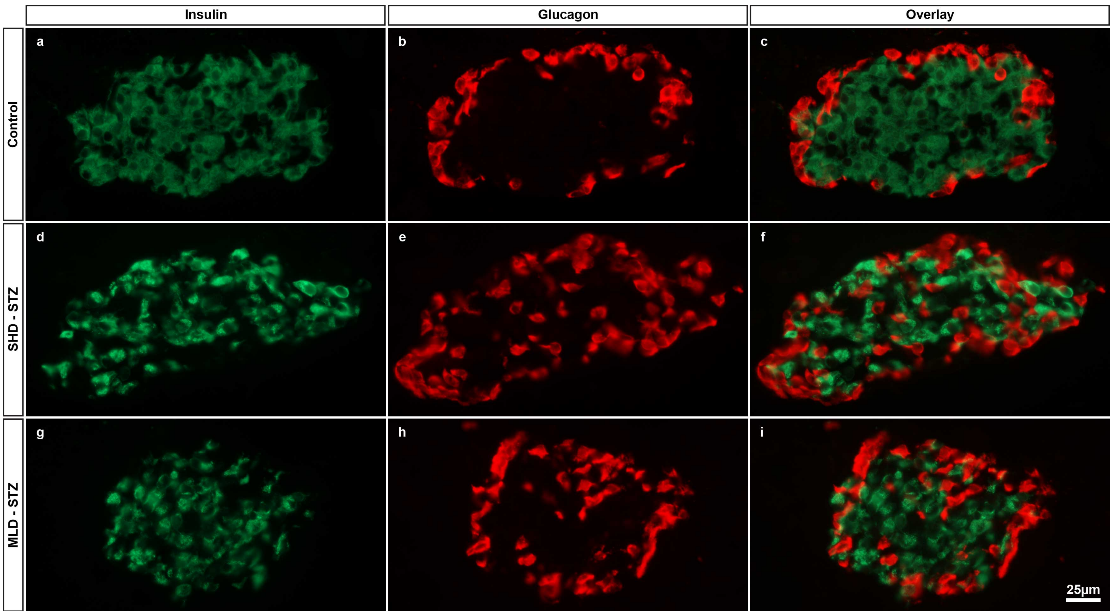

Supplementary Figure 7. The islets of STZ hyperglycemic mice consist chiefly of β-cells but have an islet core intermingled by α-cells. a-i, Representative images of sectioned islets from pancreata of control (a-c), hyperglycemic SHD-STZ (d-f) and hyperglycemic MLD-STZ (g-i) animals 3 weeks post-administration of STZ labelled for insulin (green; a, d, g,), glucagon (red; b, e, h) and insulin/glucagon (c, f, i).

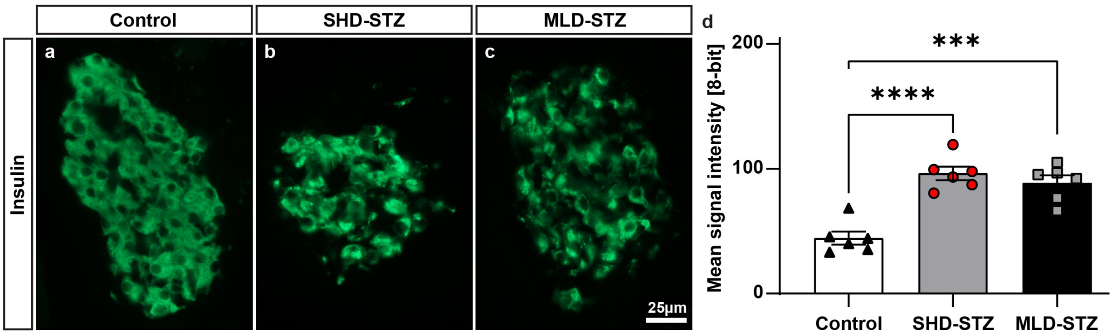

Supplementary Figure 8. Islets of STZ-treated animals display higher insulin staining intensities. a-c, Representative images of islets from control (a), SHD-STZ (b) and MLD-STZ (c) treated mice respectively, two weeks post-STZ administration. **d**, Graph displaying relative mean islet staining intensities. In each group, 6 islets sections stained for insulin were analyzed for an individual mouse, 2 weeks post-STZ administration. Data is representative of control (n=4), MLD-STZ (n=3) and SHD-STZ (n=4) mice at 1, 2 and 3 weeks post-STZ administration. Error bars represent SEM. *** and **** represent $P \leq 0.001$ and ≤ 0.0001 respectively.

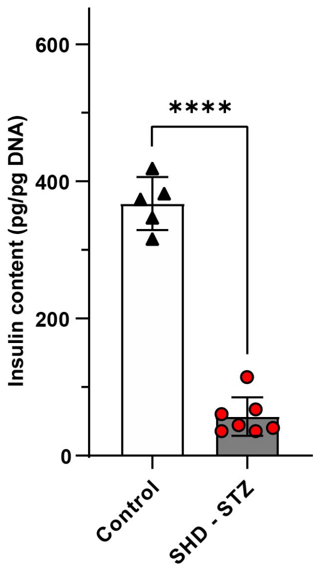

Supplementary Figure 9. STZ administration leads to a decrease in islet insulin content. Islets were isolated from mice three weeks after STZ or sham administration and lysed for measurement of insulin and DNA content. Quantification of whole islet insulin content, after normalization to DNA content, revealed a significant reduction in islets from STZ treated mice (n=5-7 mice per group). Between 8 and 10 islets were used for insulin content quantification. Data is shown as mean ± S.D. **** $p<0.0001$

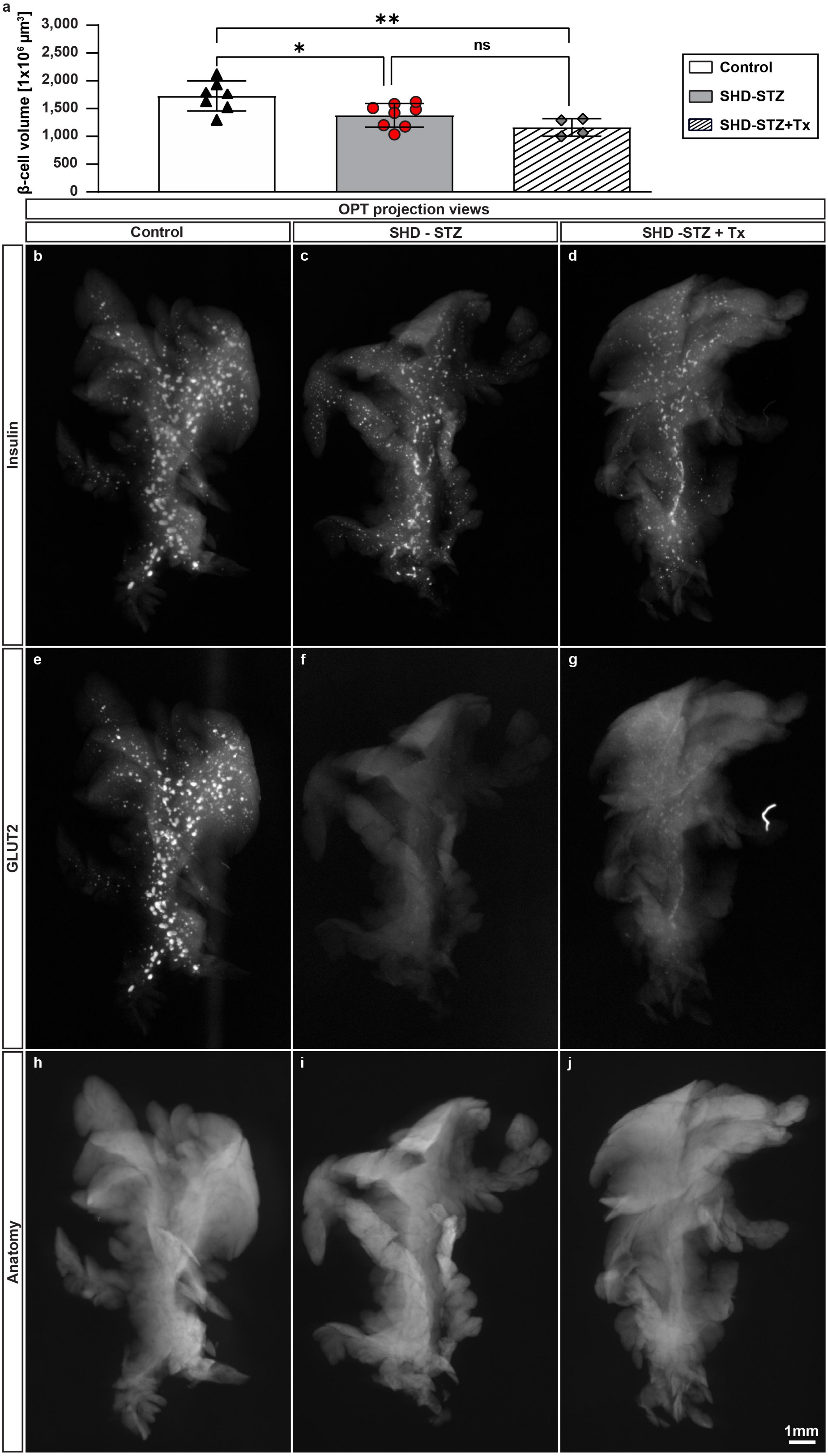
a
β-cell volume [1x10⁶ μm³]
3,000
2,500
2,000
1,500
1,000
500
0
**
*
ns
Control
SHD-STZ
SHD-STZ+Tx
OPT projection views
Control
SHD - STZ
SHD -STZ + Tx
b
c
d
Insulin
e
f
g
GLUT2
h
i
j
Anatomy
1mm

Supplementary Figure 10. GLUT2 expression in pancreata of SHD-STZ versus SHD-STZ+Tx mice. a, Bar chart displaying average β-cell volume in pancreata of vehicle controls (white, n=7), SHD-STZ-treated (gray, n=8) and SHD-STZ+Tx (i.e. transplanted with islets to recover normoglycemia (black, n=4). Re-establishment of normoglycemia did not result in increased BCM at the end point of the experiment (see also fig. 6). **b-j**, Representative OPT projection views obtained at the end point of the experiment showing insulin staining (b-d), GLUT2 staining (e-g) and tissue autofluorescence delineating the anatomical boundaries of the splenic pancreas (h-j) of control (b, e, h), SHD-STZ (c, f, i) and SHD-STZ+Tx (d, g, j) animals. Intense fluorescence on the right side in (g) is caused by a fiber which was excluded when iso-surfacing GLUT2 positive objects (see Fig. 6). Error bars represent SEM. * and ** represent $p \leq 0.05$ and $p \leq 0.01$ respectively.

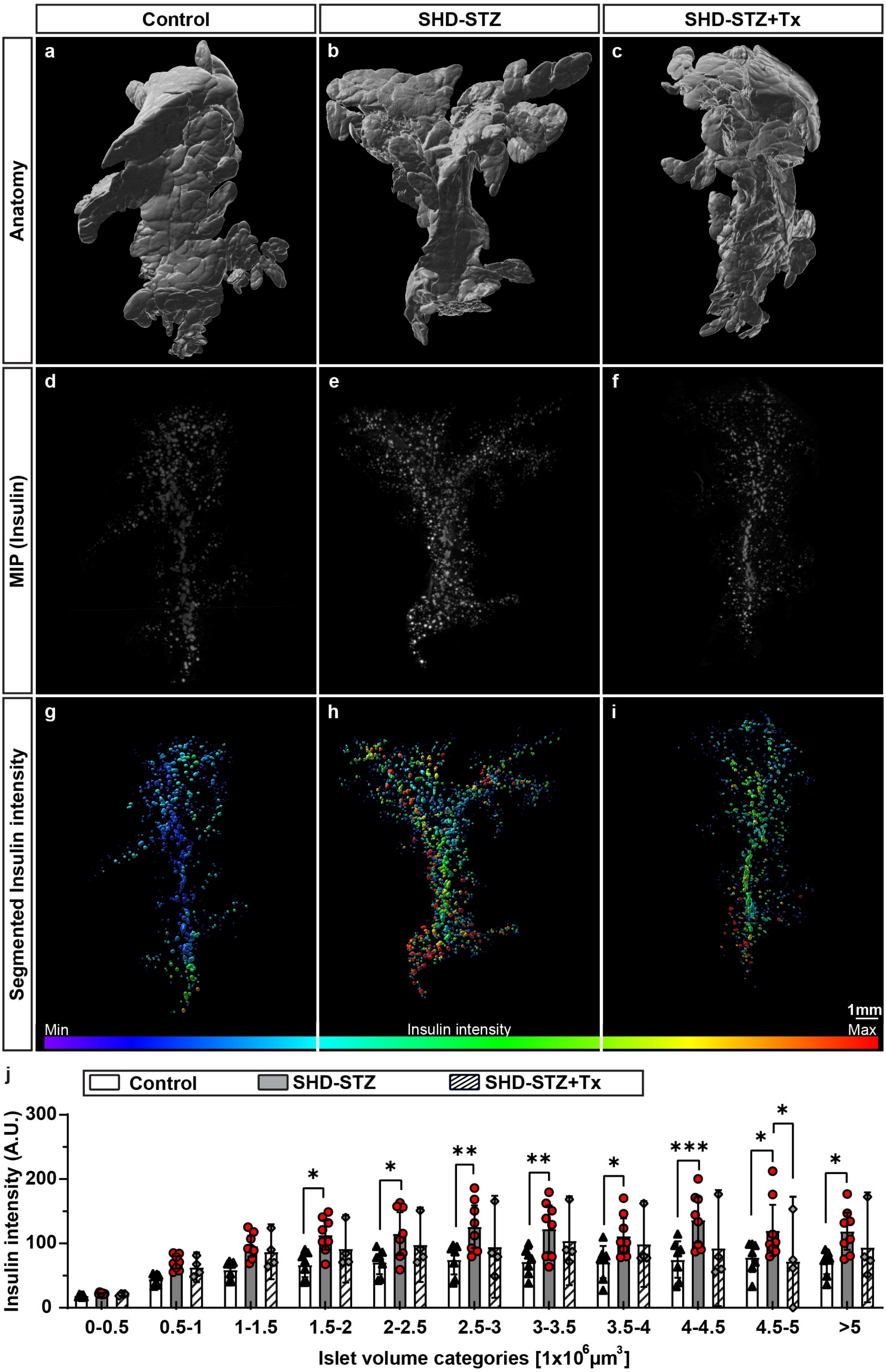

Control
SHD-STZ
SHD-STZ+Tx
Anatomy
MIP (Insulin)
Segmented Insulin intensity
a
b
c
d
e
f
g
h
i
1mm
Min
Insulin intensity
Max
j
Control
SHD-STZ
SHD-STZ+Tx
Insulin intensity (A.U.)
300
200
100
0
*
*
**
**
*
***
*
*
*
0-0.5
0.5-1
1-1.5
1.5-2
2-2.5
2.5-3
3-3.5
3.5-4
4-4.5
4.5-5
>5
Islet volume categories [1x10^6 μm^3]

Supplementary Figure 11. Insulin staining intensities are increased in SHD-STZ-treated mice. a-i, OPT images of representative pancreata (splenic lobe) from control (a, d, g), SHD-STZ (b, e, h) and SHD-STZ+Tx (c, f, i) treated animals showing segmentations of the anatomy channel displaying the outline of the gland (a-c, gray), maximum intensity projection (MIP) views of insulin staining with identical exposure times (d-f) and segmented islets color-coded for their relative signal intensities (g-i). **j**, Graph displaying insulin intensities plotted against islet volume categories. Controls (n=7, 7,294 islets), SHD-STZ (n=8, 13,627 islets) and SHD-STZ+Tx (n=4, 4,328 islets). Each replicate represents the average insulin intensity from a splenic lobe for an individual islet volume category. Error bars represent SEM. *, ** and *** represent $P \leq 0.05$, $P \leq 0.01$ and $P \leq 0.001$respectively.

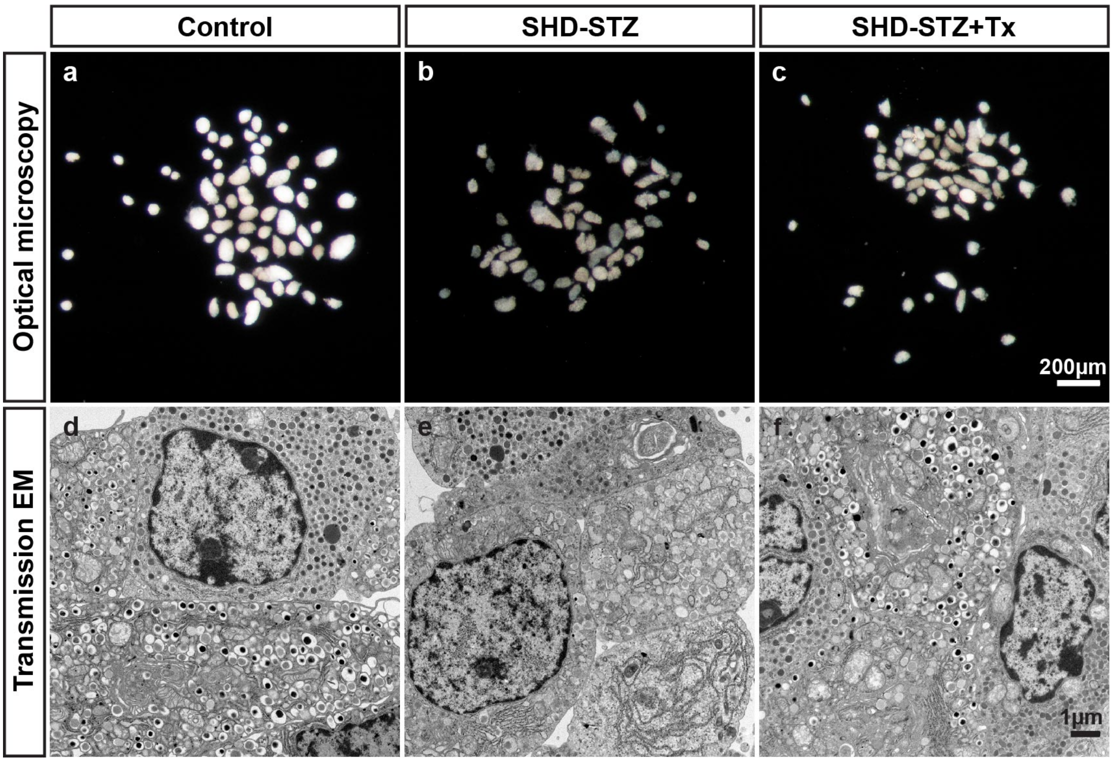

Supplementary Figure 12. Islets from STZ-treated mice display a more transparent appearance and their β-cells contain immature secretory granules. a-c, Photomicrographs of isolated islets from control (a), SHD-STZ (b) and SHD-STZ+Tx (c) animals. **d-f**, Transmission electron microscopy (TEM) images of islets from control (d), SHD-STZ (e) and SHD-STZ+Tx (f) animals.

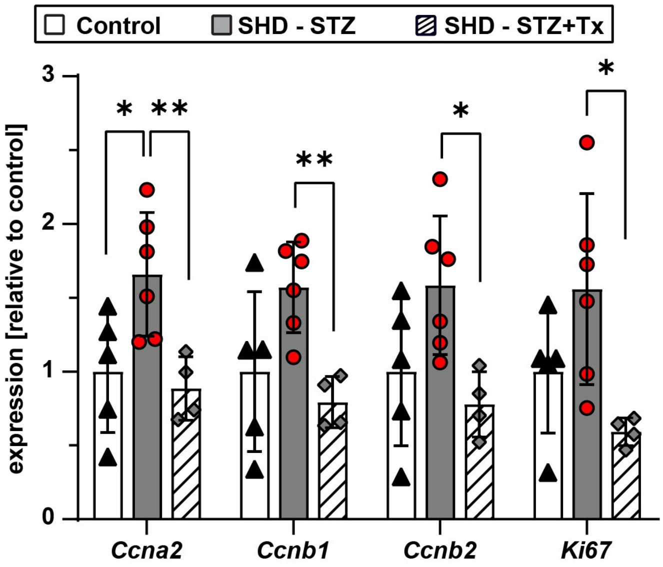

Supplementary Figure 13. β-cell proliferation is reduced in STZ-treated mice following restoration of normoglycemia. Graph displaying relative expression of genes associated with proliferation, determined by qRT-PCR of isolated islets from control, SHD-STZ and SHD-STZ+Tx mice (n=5, 6 and 4 mice for control, SHD-STZ and SHD-STZ+Tx, respectively). All samples were collected 4 weeks post-STZ administration. Error bars represent SD. * and ** represent $P \leq 0.05$ and $P \leq 0.01$ respectively.

SUPPLEMENTARY TABLES

Gene	Primer sequences	Ref
Aldh1a3	5’- GGG TCA CAC TGG AGC TAG GA -3’<br>5’- CTG GCC TCT TCT TGG CGA A -3’	(1)
Ccna2	5’- GCC TTC ACC ATT CAT GTG GAT -3’<br>5’- TTG CTG CGG GTA AAG AGA CAG -3’	(2)
Ccnb1	5’- GCG TGT GCC TGT GAC AGT TA -3’<br>5’- CCT AGC GTT TTT GCT TCC CTT -3’	(2)
Ccnb2	5’- AGC TCC CAA GGA TCG TCC TC -3’<br>5’- TGT CCT CGT TAT CTA TGT CCT CG -3’	(2)
Ins1	5’- TAG TGA CCA GCT ATA ATC AGA G -3’<br>5’- ACG CCA AGG TCT GAA GGT CC	(3)
Ins2	5’- TCT ACA CAC CCA TGT CCC GC -3’<br>5’- ACA ATG CCA CGC TTC TGC TG -3’	
G6pc2	5’- CTG GTC CTT TCT GTG GAG TGT -3’<br>5’- TCC AAG AAT GAC CTG ATG GGG -3’	(4)
Gck	5’- CAA CTG GAC CAA GGG CTT CAA -3’<br>5’- TGT GGC CAC CGT GTC ATT C -3’	
Glp1r	5’- GGG TCT CTG GCT ACA TAA GGA CAA C -3’<br>5’- AAG GAT GGC TGA AGC GAT GAC -3’	(5)
Glut2	5’- ACC CTG TTC CTA ACC GGG -3’<br>5’- TGA ACC AAG GGA TTG GAC C -3’	(6)
Ki67	5’- TTG ACC GCT CCT TTA GGT ATG AA -3’<br>5’- TTC CAA GGG ACT TTC CTG GA -3’	(7)
Pdx1	5’- CTT AAC CTA GGC GTC GCA CAA -3’<br>5’- GAA GCT CAG GGC TGT TTT TCC -3’	(2)
Mafa	5’- CAG CAG CGG CAC ATT CTG -3’<br>5’- GCC CGC CAA CTT CTC GTA T -3’	(8)
Nkx6.1	5’- CTC TAC TTT AGC CCC AGC G -3’<br>5’- CAC GGC GGA CTC TGC ATC ACT C -3’	
Serpina7	5’- TCC CCT GTG AGC ATA TCT GTT -3’<br>5’- CGG GAG TAT CTG TGA GGT TAA AC -3’	(9)
Slc30a8	5’- CAG AGA ACT TCG ACA GAA GCC -3’<br>5’- CTT GCT TGC TCG ACC TGT T -3’	(2)
Tbp	5’- TGC TGT TGG TGA TTG TTG GT -3’<br>5’- CTG GCT TGT GTG GGA AAG AT -3’	(10)
Trpm5	5’ TGC TCA AGG GTA CCC AAT GCT ACT -3’<br>5’- CCG GGT GTT TGA AAT GTC CCG TTT -3’	(11)
Ucn3	5’- GCT GTG CCC CTC GAC CT -3’<br>5’- TGG GCA TCA GCA TCG CT -3’	(2)

Supplementary Table 1. Primers used for qRT-PCR experiments. Primers not designed for this study are from the publications listed in Supplementary References.

SUPPLEMENTARY REFERENCES

1. J. S. Stancill, J.-P. Cartailler, H. W. Clayton, J. T. O'Connor, M. T. Dickerson, P. K. Dadi, A. B. Osipovich, D. A. Jacobson, M. A. Magnuson, Chronic β-Cell Depolarization Impairs β-Cell Identity by Disrupting a Network of Ca^{2+}-Regulated Genes, *Diabetes* **66**, 2175–2187 (2017).

2. B. L. Taylor, F.-F. Liu, M. Sander, Nkx6.1 is essential for maintaining the functional state of pancreatic beta cells, *Cell Rep* **4**, 1262–1275 (2013).

3. A. Kubo, R. Stull, M. Takeuchi, K. Bonham, V. Gouon-Evans, M. Sho, M. Iwano, Y. Saito, G. Keller, R. Snodgrass, M. Pera, Ed. Pdx1 and Ngn3 Overexpression Enhances Pancreatic Differentiation of Mouse ES Cell-Derived Endoderm Population, *PLoS ONE* **6**, e24058–12 (2011).

4. C. K. Wong, A. K. Wade-Vallance, D. S. Luciani, P. K. Brindle, F. C. Lynn, W. T. Gibson, The p300 and CBP Transcriptional Coactivators Are Required for β-Cell and α-Cell Proliferation, *Diabetes* **67**, 412–422 (2018).

5. G. Xu, H. Kaneto, D. R. Laybutt, V. F. Duvivier-Kali, N. Trivedi, K. Suzuma, G. L. King, G. C. Weir, S. Bonner-Weir, Downregulation of GLP-1 and GIP receptor expression by hyperglycemia: possible contribution to impaired incretin effects in diabetes, *Diabetes* **56**, 1551–1558 (2007).

6. E. Stolarczyk, M. Le Gall, P. Even, A. Houllier, P. Serradas, E. Brot-Laroche, A. Leturque, Loss of sugar detection by GLUT2 affects glucose homeostasis in mice, *PLoS ONE* **2**, e1288 (2007).

7. A. Klochendler, N. Weinberg-Corem, M. Moran, A. Swisa, N. Pochet, V. Savova, J. Vikeså, Y. Van de Peer, M. Brandeis, A. Regev, F. C. Nielsen, Y. Dor, A. Eden, A transgenic mouse marking live replicating cells reveals in vivo transcriptional program of proliferation, *Dev. Cell* **23**, 681–690 (2012).

8. Y. Sato, M. Inoue, T. Yoshizawa, K. Yamagata, T. Minamino, Ed. Moderate hypoxia induces β-cell dysfunction with HIF-1-independent gene expression changes, *PLoS ONE* **9**, e114868 (2014).

9. PrimerBank: https://pga.mgh.harvard.edu/primerbank/index.html

10. T. Farfel-Becker, E. Vitner, H. Dekel, N. Leshem, I. B. Enquist, S. Karlsson, A. H. Futerman, No evidence for activation of the unfolded protein response in neuronopathic models of Gaucher disease, *Human Molecular Genetics* **18**, 1482–1488 (2009).

11. Y. Asai, J. R. Holt, G. S. G. Géléoc, A quantitative analysis of the spatiotemporal pattern of transient receptor potential gene expression in the developing mouse cochlea, *J. Assoc. Res. Otolaryngol.* **11**, 27–37 (2010).
