# Supplementary material for: Topologically selective islet vulnerability and self-sustained downregulation of markers for β-cell maturity in streptozotocin-induced diabetes
Source: Commun Biol. 2020 Sep 30;3:541. doi: 10.1038/s42003-020-01243-2 (PMC7527346; doi:10.1038/s42003-020-01243-2)
Supplement: Supplementary file 2 — Description of Additional Supplementary Files [file 42003_2020_1243_MOESM2_ESM.pdf]

Description of Additional Supplementary Files

Supplementary Movie 1.
Movie of a representative splenic pancreatic lobe of control animal imaged by OPT. Islet β-cell volumes (iso-surfaced) are pseudo coloured to delineate small (<1 x10^6 µm^3, white), intermediate (1-5 x10^6 µm^3, red) and large (>5 x10^6 µm^3, yellow) islets. The anatomical volume of the lobe is displayed in grey.

Supplementary Movie 2.
Movie of a representative splenic pancreatic lobe of a SHD-STZ treated animal, three weeks post STZ administration, imaged by OPT. Islet β-cell volumes (iso-surfaced) are pseudo coloured to delineate small (<1 x10^6 µm^3, white), intermediate (1-5 x10^6 µm^3, red) and large (>5 x10^6 µm^3, yellow) islets. The anatomical volume of the lobe is displayed in grey.

Supplementary Movie 3.
Movie of a representative splenic pancreatic lobe of a MLD-STZ treated animal, three weeks post STZ administration, imaged by OPT. Islet β-cell volumes (iso-surfaced) are pseudo coloured to delineate small (<1 x10^6 µm^3, white), intermediate (1-5 x10^6 µm^3, red) and large (>5 x10^6 µm^3, yellow) islets. The anatomical volume of the lobe is displayed in grey.

Supplementary Movie 4.
Light sheet fluorescence microscopy movie (maximum projection intensity) displaying islets of Langerhans labeled for insulin (red) in the central region of the splenic pancreatic lobe of a control animal.

Supplementary Movie 5.
Light sheet fluorescence microscopy movie (maximum projection intensity) displaying islets of Langerhans labeled for insulin (red) in the central region of the splenic pancreatic lobe of a SHD-STZ treated animal.

Supplementary Movie 6.
Light sheet fluorescence microscopy movie (maximum projection intensity) displaying islets of Langerhans labeled for insulin (red) in the central region of the splenic pancreatic lobe of a MLD-STZ treated animal.

Supplementary movie 7.
Movie of a representative splenic pancreatic lobe of a control animal imaged by OPT.
Islets labelled for insulin (red) and GLUT2 (blue) are displayed both as maximum intensity projections (MIP) and as iso-surfaced volumes. Regions were the two channels overlap are shown in yellow (MIP and iso-surface, respectively). The anatomical volume of the pancreatic lobe is displayed in grey.

Supplementary movie 8.
Movie of a representative splenic pancreatic lobe of a SHD-STZ treated animal imaged by OPT. Islets labelled for insulin (red) and GLUT2 (blue) are displayed both as maximum intensity projections (MIP) and as iso-surfaced volumes. Regions were the two channels overlap are shown in yellow (MIP and iso-surface, respectively). The anatomical volume of the pancreatic lobe is displayed in grey.

Supplementary movie 9.
Movie of a representative splenic pancreatic lobe of a SHD-STZ+Tx treated animal imaged by OPT. Islets labelled for insulin (red) and GLUT2 (blue) are displayed both as maximum intensity projections

(MIP) and as iso-surfaced volumes. Regions were the two channels overlap are shown in yellow (MIP and iso-surface, respectively). The anatomical volume of the pancreatic lobe is displayed in grey. An artefact in the form of a textile fibre is visible in both the insulin and GLUT2 channel.

Supplementary data 1
Individual data points corresponding to figures 1b, 1c, 2a, 2b, 2c, 3a, 3b, 6g, 7a, 7c, 7d and 7e.
