# Supplementary material for: Topologically selective islet vulnerability and self-sustained downregulation of markers for β-cell maturity in streptozotocin-induced diabetes
Source: Commun Biol. 2020 Sep 30;3:541. doi: 10.1038/s42003-020-01243-2 (PMC7527346; doi:10.1038/s42003-020-01243-2)
Supplement: Supplementary file 13 — Reporting Summary [file 42003_2020_1243_MOESM13_ESM.pdf]

Corresponding author(s): Erwin Ilegems
Ulf Ahlgren

Last updated by author(s): Jul 18, 2020

Reporting Summary

Nature Research wishes to improve the reproducibility of the work that we publish. This form provides structure for consistency and transparency in reporting. For further information on Nature Research policies, see Authors & Referees and the Editorial Policy Checklist.

Statistics

For all statistical analyses, confirm that the following items are present in the figure legend, table legend, main text, or Methods section.

n/a	Confirmed	
☐	☒	The exact sample size (*n*) for each experimental group/condition, given as a discrete number and unit of measurement
☐	☒	A statement on whether measurements were taken from distinct samples or whether the same sample was measured repeatedly
☐	☒	The statistical test(s) used AND whether they are one- or two-sided<br>*Only common tests should be described solely by name; describe more complex techniques in the Methods section.*
☒	☐	A description of all covariates tested
☐	☒	A description of any assumptions or corrections, such as tests of normality and adjustment for multiple comparisons
☐	☒	A full description of the statistical parameters including central tendency (e.g. means) or other basic estimates (e.g. regression coefficient) AND variation (e.g. standard deviation) or associated estimates of uncertainty (e.g. confidence intervals)
☒	☐	For null hypothesis testing, the test statistic (e.g. *F*, *t*, *r*) with confidence intervals, effect sizes, degrees of freedom and *P* value noted<br>*Give P values as exact values whenever suitable.*
☒	☐	For Bayesian analysis, information on the choice of priors and Markov chain Monte Carlo settings
☒	☐	For hierarchical and complex designs, identification of the appropriate level for tests and full reporting of outcomes
☒	☐	Estimates of effect sizes (e.g. Cohen's *d*, Pearson's *r*), indicating how they were calculated

Our web collection on statistics for biologists contains articles on many of the points above.

Software and code

Policy information about availability of computer code

Data collection	Image data was acquired using in-house developed NIR-OPT software (Eriksson A. U., Svensson, C. J. Vis. Exp., 2013, https://doi.org/10.3791/50238), BiOPTonics SkyScanner 3001 (version 1.3.13, SkyScan), ImSpectorPro Data Acquisition and Analysis Environment (version 5.0.164, Lavision Biotec GmbH), NIS Elements F (version 3.22.00, Nikon), Leica Application Suite Advanced Fluorescence Software (LAS AF 2.7.6, Leica Microsystems) and Veleta camera (Olymous Soft Imaging, GmbH).
Data analysis	OPT data processing was performed using in-house generated software for i) COM-AR (i.e. semi-automatic positioning of the samples at the axis of rotation), ii) determination of post-alignment values (as described in Cheddad et al., IEEE transaction on medical imaging, 2012 https://doi.org/10.1109/TMI.2011.2161590) and for iii) CLAHE (contrast limited adaptive histogram equalisation (as described in Hörnblad et al., Islets, 2011, https://doi.org/10.4161/isl.3.4.16417). These scripts are available upon request to the authors subject to a material transfer agreement (MTA). Further, the dynamic range of the projection images was adjusted using DataViewer (version 1.5.6.2, Bruker microCT), to facilitate reconstruction. Tomographic reconstruction of projection images was performed using NRecon (implementing the Feldkamp algorithm for cone-beam geometry, version 1.6.9.18 SkyScan). Imaris File Converter (batch conversion from reconstructed tomographic sections to Imaris files, version 9.3.0, Bitplane) was used to convert reconstructed *.bmp files to the Imaris native format *.ims. Measurements of islet volumes and intensities were performed in Imaris (version 9.3.1, Bitplane AG). Confocal image stacks were processed with Fiji (version 1.50d, J Schindelin et al. Nature Methods, 2012) using the Interactive Stack Rotation plugin. Staining intensities on IHC sections was acquired using Volocity (PerkinElmer). Adobe Photoshop CS6 (version 13.0, Adobe) and Adobe Illustrator CS6 (version 16.0.0, Adobe) were used for figure assembly and contrast enhancement of representative images. Parameters for individual samples regarding dynamic range adjustments, reconstruction, Imaris thresholding etc., listed above, are available upon request to the authors. Excel (version 1908, Microsoft) and GraphPad Prism (version 8.2.1, GraphPad Software, Inc.) were used for statistical analysis.

For manuscripts utilizing custom algorithms or software that are central to the research but not yet described in published literature, software must be made available to editors/reviewers. We strongly encourage code deposition in a community repository (e.g. GitHub). See the Nature Research guidelines for submitting code & software for further information.

Data

Policy information about availability of data

All manuscripts must include a data availability statement. This statement should provide the following information, where applicable:

- Accession codes, unique identifiers, or web links for publicly available datasets
- A list of figures that have associated raw data
- A description of any restrictions on data availability

The data (including OPT projection views, tomographic image stacks and Light sheet fluorescence data) that support the findings of this study constitute around 3 Terabytes. This data (or parts thereof) is available from the corresponding author upon reasonable request.

Field-specific reporting

Please select the one below that is the best fit for your research. If you are not sure, read the appropriate sections before making your selection.

☒ Life sciences ☐ Behavioural & social sciences ☐ Ecological, evolutionary & environmental sciences

For a reference copy of the document with all sections, see nature.com/documents/nr-reporting-summary-flat.pdf

Life sciences study design

All studies must disclose on these points even when the disclosure is negative.

Sample size	OPT sample size calculation was performed based on previous studies on diabetic mouse models (T. Alanentalo, et al., Nat Methods, 2007 and S. Parween et al. Scientific Reports, 2016). Note, by nature of the tomographic technique used, the generated data sets are, at least in theory, “absolute”, i.e. all islets of each pancreas are included in the data sets (translating to about 5000 islets of Langerhans per control pancreas). In contrast, commonly utilized stereological sampling techniques estimate islets mass, number etc. based on a limited number of islets (normally only a few hundred). For transplantation experiments, sample size was determined based on previously performed experiments (Van Krieken et al., Scientific Reports, 2017).
Data exclusions	For longitudinal in vivo imaging, a few individual islet grafts were excluded from analysis when overgrowth of the transplant by iris-derived cells from the host mouse prevented further measurements of islet dimensions. For studies of β-cell mass in SHD-STZ and MLD-STZ hyperglycemic mice, animals that did not display display blood glucose concentration >12mmol/L were excluded from the analysis.
Replication	Immunohistochemical experimental finding on sections were repeated at least twice with reproducible results. Glucose measurements were always performed in duplicate. The reproducibility of OPT-based assessments of BCM has previously been established (see e.g. Alanentalo et al., Nature Methods 2007, Hörnblad et al., Islets 2011).
Randomization	All samples were randomized after organ harvest for OPT-processing, OPT-image acquisition and data analysis. Regarding transplantation experiments, all mice were randomly allocated into their experimental group except for the mice receiving curative transplantation in the "recovery experiment". As mentioned in the manuscript, these mice had the highest blood glucose four days after STZ treatment and would likely have suffered too much weight loss during the duration of the experiment if left untransplanted.
Blinding	All samples were blinded to the investigator after organ harvest for OPT analyses. For longitudinal in vivo imaging experiments, blinding was not possible as blood glucose and islet volume measurements were unequivocally different between STZ-treated and control mice.

Reporting for specific materials, systems and methods

We require information from authors about some types of materials, experimental systems and methods used in many studies. Here, indicate whether each material, system or method listed is relevant to your study. If you are not sure if a list item applies to your research, read the appropriate section before selecting a response.

Materials & experimental systems

n/a	Involved in the study	
☐	☒	Antibodies
☒	☐	Eukaryotic cell lines
☒	☐	Palaeontology
☐	☒	Animals and other organisms
☒	☐	Human research participants
☒	☐	Clinical data

Methods

n/a	Involved in the study	
☒	☐	ChIP-seq
☒	☐	Flow cytometry
☒	☐	MRI-based neuroimaging

Antibodies

Antibodies used	Primary Antibodies:

Polyclonal; guinea pig anti-insulin (A0564 DAKO, lot# 10042817),
rabbit anti-GLUT2 (07-1402-I Millipore, lot# 2987671),
rabbit anti-glucagon (20076 Immunostar, lot# 1715001),
rabbit anti Cleaved Caspase3 (Cell Signaling, 9661, dilution 1:500),
rabbit anti MafA (Bethyl, A300-611A-3, dilution 1:250)
rabbit anti PDX-1 (Helena Edlund lab own stock, dilution 1:500)
Secondary Antibodies:
goat Alexa Fluor 594 anti-guinea pig IgG (A11076 Molecular Probes, lot# 1924784),
goat IRDye 680RD anti-rabbit IgG (926-68071 LI-COR, lot# C80911-15),
goat Alexa Fluor 594 anti-rabbit IgG (A11012, Molecular Probes, lot# 1515530),
goat Alexa Fluor 488 anti-guinea pig (A11073 Molecular Probes, lot# 1458631),

Validation	Antibody validation information for the antibodies utilized in the study is available from each manufacturer under their respective catalogue number. In addition, all antibodies underwent a standardized in-house titration on sections with pancreatic mouse tissue and were validated for cross reactivity before being used in whole mount the immunohistochemical analyses.

Animals and other organisms

Policy information about studies involving animals; ARRIVE guidelines recommended for reporting animal research

Laboratory animals	For all OPT imaging experiments, 8-weeks-old male C57BL/6J mice were used. For islet isolation (and associated experiments), male and female mice aged 8-12 weeks on a C57BL/6J background were used. Animals were purchased from Charles River and housed at the Umeå Centre for Comparative Biology and at the animal facilities at Karolinska Institutet.
Wild animals	The study did not involve wild animals.
Field-collected samples	The study did not involve samples collected from the field.
Ethics oversight	All experiments were performed following the European Union guidelines for care and use of animals in research, and all procedures were approved by the Animal Review Board at the Court of Appeal of Northern Norrland and of Northern Stockholm.

Note that full information on the approval of the study protocol must also be provided in the manuscript.
